# Chemokine Receptors—Structure-Based Virtual Screening Assisted by Machine Learning

**DOI:** 10.3390/pharmaceutics15020516

**Published:** 2023-02-03

**Authors:** Paulina Dragan, Matthew Merski, Szymon Wiśniewski, Swapnil Ganesh Sanmukh, Dorota Latek

**Affiliations:** Faculty of Chemistry, University of Warsaw, 02-093 Warsaw, Poland

**Keywords:** cheminformatics, machine learning, neural network, gradient-boosting machine, molecular docking, virtual screening, G protein-coupled receptors, chemokine receptors, CCR2, CCR3, drug discovery, TensorFlow, LightGBM, Glide

## Abstract

Chemokines modulate the immune response by regulating the migration of immune cells. They are also known to participate in such processes as cell–cell adhesion, allograft rejection, and angiogenesis. Chemokines interact with two different subfamilies of G protein-coupled receptors: conventional chemokine receptors and atypical chemokine receptors. Here, we focused on the former one which has been linked to many inflammatory diseases, including: multiple sclerosis, asthma, nephritis, and rheumatoid arthritis. Available crystal and cryo-EM structures and homology models of six chemokine receptors (CCR1 to CCR6) were described and tested in terms of their usefulness in structure-based drug design. As a result of structure-based virtual screening for CCR2 and CCR3, several new active compounds were proposed. Known inhibitors of CCR1 to CCR6, acquired from ChEMBL, were used as training sets for two machine learning algorithms in ligand-based drug design. Performance of LightGBM was compared with a sequential Keras/TensorFlow model of neural network for these diverse datasets. A combination of structure-based virtual screening with machine learning allowed to propose several active ligands for CCR2 and CCR3 with two distinct compounds predicted as CCR3 actives by all three tested methods: Glide, Keras/TensorFlow NN, and LightGBM. In addition, the performance of these three methods in the prediction of the CCR2/CCR3 receptor subtype selectivity was assessed.

## 1. Introduction

Chemokines, also referred to as chemotactic cytokines, are small proteins that regulate the migration of immune cells through the activation of G protein-coupled receptors (GPCRs) [1]. Chemokines can be classified by the arrangement of the N-terminal cysteines responsible for the creation of disulfide bridges. There are four different chemokine families: CXC, CC, XC, and CX3C, where X is any amino acid other than cysteine [2]. Based on their function, they can also be divided into homeostatic, inflammatory, and dual homeostatic/inflammatory chemokines [2]. The first group are chemokines expressed in homeostatic conditions that take part in the transport of non-effector leukocytes [3]. Inflammatory chemokines, on the other hand, are produced by leukocytes and other cells mostly in response to tissue damage [4] or infection [1]. They are responsible for the chemotaxis of leukocytes to inflamed or injured areas [2]. In addition to cell movement, chemokines participate in: angiogenesis, cell–cell adhesion, embryonic development, integrin regulation, and protease secretion, among others [5]. Chemokines also play a role in numerous pathological processes, e.g., infection, inflammation, allergies, autoimmune and vascular diseases, neoplasia, and allograft rejection [6].

Two main families of chemokine receptors have been so far recognized: conventional chemokine receptors and atypical chemokine receptors [5]. Both consist of seven transmembrane domains that are located within the cell membrane [1]. Many chemokines can bind to multiple receptors; the same is true for their receptors, i.e., some of them can bind multiple chemokines—this promiscuity is often observed in relation to inflammatory processes [5]. Conventional chemokine receptors (cCKRs) can be grouped depending on which chemokine subfamilies they bind: CXCR (6 receptors [7]), CCR (10 receptors [7]), XCR (1 receptor [7]), and CX3CR (1 receptor [7]) [1]. As GPCRs, chemokine receptors interact with G proteins and β-arrestins during signal transduction and demonstrate biased signaling if bound to functionally selective ligands. According to the two-site model of the chemokine binding, extracellular loops and N-terminus of cCKRs are responsible for the initial interactions with chemokines, while N-terminus of chemokines induces the receptor activation [5]. This model, however, has been suggested to be oversimplified and does not take into account other important ligand-receptor interactions [8]. A list of ligands that bind to a certain subtype of CC chemokine receptors, as well as to the corresponding immune cell subset, is presented in Table 1.

Four atypical chemokine receptors (ACKRs) have been discovered to date. Despite their structures being reminiscent of cCKRs, atypical receptors are not coupled with G proteins and therefore do not participate in conventional cellular signaling. Due to this, they are sometimes referred to as “silent” receptors [15]. ACKRs have been shown to function as decoy and/or scavenger receptors [16], i.e., they bind chemokines in order to reduce their number and thus prevent activation [17]. At present, most anti-inflammatory drugs target leukocytes. However, because of the role they play in the immune response and inflammation, chemokine receptors have been identified as potentially more efficient drug targets [11]. Conventional CKRs have been shown to be involved in the pathology of numerous diseases. Drugs targeting chemokine receptors include but are not limited to:

- maraviroc, a CCR5 antagonist used to inhibit the entry of the human immunodeficiency virus (HIV) into cells by introducing steric hindrance. HIV hijacks CCR5 [18], and to a lesser extent CXCR4, in order to access human cells [5];
- plerixafor and mavorixafor, both of which target CXCR4. Plerixafor is used for the mobilization of hematopoietic stem cells [19], and mavorixafor is in phase III clinical trials [20];
- vercirnon, a CCR9 antagonist that is currently in phase III clinical trials and has the potential to treat Crohn’s disease, celiac disease, and ulcerative colitis [21].

There are certain hurdles that need to be overcome to utilize chemokine receptors as drug targets for inflammation, e.g., inadequate in vivo dosing to inhibit the receptor activity. The promiscuity of this system also raises concerns, though it has been suggested that this may be overexaggerated, as structural, generic, and pharmacological evidence suggest that there is no redundancy in the chemokine system. Inappropriate target selection seems to be a much more plausible reason for difficulties in utilizing chemokine receptors in pharmacotherapy [22].

An initial connection between CC chemokines (β-chemokines) and viral infections was made with the discovery that RANTES, macrophage inflammatory proteins MIP-1α, and MIP-1β (also known as CCL5, CCL3, and CCL4) effectively suppress HIV-1 [23,24]. This suggested that these molecules may control the immune response to infection, and that sustained delivery of the respective receptor inhibitors could result in long-term control of infection [23,24,25]. It is likely that these molecules play a positive role in controlling the natural course of HIV infection since chemokine production is associated with antigen-induced proliferative responses. This results in a better clinical status of HIV patients and a decreased probability of infection in at-risk subjects [23,24,25]. Besides HIV, a plethora of different viruses induce CC chemokine expression in humans and other species during infections, as shown in Table 2. This justifies the usage of chemokine receptors as drug targets in various infectious diseases.

Among CC chemokines, CCL2 (MCP-1) and CCL3 (MIP-1α) are involved in the immune response to SARS-CoV-2 [44]. CXC chemokines, such as CXCL5, CXCL9, and CXCL10 have been studied concerning COVID-19 as potential biomarkers for this disease [45]. Immunosuppressing therapies for COVID-19 were described in [46], while Mehta et al. investigated immunosuppression in severe conditions involving the cytokine storm syndrome [47].

As was mentioned above, drug discovery for chemokine receptors still encounters many difficulties [22] but there have also been many successes [48]. Recently, the use of ACKRs as anti-cancer drug targets is an emerging therapeutic direction [49]. In general, drug discovery is a long, difficult, and expensive process, frequently ending with drug withdrawal at the clinical or even post-clinical phase. For this reason, drug repositioning is frequently used to limit both the cost and the number of failures [50]. Computational methods, including machine learning or artificial intelligence, can be also used to significantly reduce the initial discovery costs [51,52]. Prediction models that utilize machine learning include, e.g., Glmnet, XGBoost, or LightGBM [53]. LightGBM (Light Gradient Boosting Machine) is based on the Gradient Boosting Decision Tree (GBDT) algorithm, and implements gradient-based one-side sampling (GOSS) and exclusive feature bundling (EFB) [54].

Here, we trained a sequential neural network prepared with Keras API integrated with TensorFlow [55] and a gradient boosting machine implemented in LightGBM [54] with datasets including active ligands of chemokine receptors. Thus, their applicability in ligand-based drug design could be assessed [56,57]. In addition, the currently available CCR1 through CCR6 crystal and cryo-EM structures and homology models were evaluated in terms of their usefulness in structure-based virtual screening (SBVS) following our previous studies on class B receptors (GCGR, GLP-1R [58], VIP and PACAP receptors [50]). For two CC receptors (CCR2 and CCR3) SBVS was performed against the Enamine screening collection (HLL-460) including diverse chemotypes [59] to search for novel inhibitors and new active-like scaffolds.

## 2. Materials and Methods

### 2.1. Crystal/Cryo-EM Structures and Models of Chemokine Receptors

The study involved the following receptors: CCR1, CCR2, CCR3, CCR4, CCR5, and CCR6. At the time, only three of them had structures deposited in PDB, and of these only CCR5 had structures of both the active and inactive receptor states. For this reason, models were created of the missing active, inactive, and intermediate structures—this was performed using GPCRdb [60], I-TASSER [61,62,63], and Robetta [64]. Every generated structure was analyzed using PyMOL [65], and those that were assumed to be the highest quality were selected to be used in further steps. Both the PDB structures and homology models are listed in Table 3.

The receptor structures were imported into Maestro, Schrodinger LLC [75], where they were preprocessed using the Protein Preparation Wizard with the default settings. All water molecules, metal ions, and ligands not in the active site were removed; the remaining ligand was then split from the receptor to prepare both structures for molecular docking. Low-energy structures of the ligand were obtained using LigPrep [76] with the default settings. The protonation state of each compound was determined with Epik (Schrodinger, LLC). In the case of peptide or small protein (chemokine) ligands in the PDB structure, the ligand was truncated to ca. 10 residues to allow sufficient sampling of its conformational space during Glide docking. Glide [77] was then used to generate a receptor grid and dock the lowest energy ligand conformation onto the receptor. The docking scores of the ligand poses were recorded and heavy-atom RMSD was calculated (Schrodinger, LLC, script *rmsd.py*) in order to determine the extent to which the ligand position had changed in regards to that in the original PDB structure. If more than one PDB structure of the receptor existed, cross-docking was performed to check the docking scores and RMSD in relation to the other structures, and thereby determine the structure quality in terms of structure-based VS. This was done for all three CCR2 structures and the ligands from four different CCR5 structures. In addition, second, more exact cross-docking was performed for three of the CCR5 structures due to the significant similarities between their ligands. The obtained docking scores and RMSD values were compared between receptors.

The selected receptor models were imported into Maestro [75], where they were preprocessed for the enrichment study. ChEMBL [78,79] was searched in order to find ligands known to interact with these receptors (actives). A total of 100 ligands were chosen for each receptor and used to prepare a set of decoys using DUD-E [80], as was conducted previously for class B receptor ligands [58]. Actives and decoys were combined to form test sets, which were then prepared with LigPrep and docked onto every CC receptor. ROC (receiver operating characteristic) curves [58] were created in order to determine the ability of the models to differentiate between actives and decoys.

### 2.2. Structure-Based Virtual Screening Involving CCR2/CCR3 Receptor Subtype Selectivity

Structure-based virtual screening was performed using the inactive-state 6GPX crystal structure of CCR2 and the inactive-state Robetta model of CCR3. The Hit Locator Library (HLL-460), downloaded from https://enamine.net/ [59] on 17 November 2022 and consisting of 460,160 compounds that encompass the website’s entire screening collection of diverse chemotypes, was used. Maestro’s Canvas Similarity and Clustering was used to cluster the compounds of the best docking scores. The chosen similarity metric was Tanimoto [81], and the average linkage method was applied. Medoid compounds in each of ca. 20 clusters were selected for analysis.

### 2.3. Ligand-Based Drug Design for CCR1–6

Active compounds for receptors CCR1–6 were downloaded from the ChEMBL database [79] in September 2022. The human version of each receptor with the largest number of ligands was selected and the subset including mostly the IC50 sub-category was downloaded (see Supplementary Tables S1 and S2). Among these compounds, no allosteric compounds (except one compound in the CCR2 dataset) were found by text mining. Compounds that were duplicated were removed from datasets. Based on SMILES—the numerically coded descriptor, Morgan fingerprints (ECFP4) [82] were then generated using Pandas (v. 1.5). Compounds were then subdivided into categories by the logarithmic-scale activity values (pChEMBL) that corresponded to IC50 (half-maximal inhibitory concentration). Compounds demonstrating inhibitory activity for the target at the level greater than 100 µM (pChEMBL values < 4.0) were considered as ‘inactives’ (ligands tested experimentally to be inactive ones). For example, in the CCR5, 6% of the compounds were in the low activity range of pChEMBL values 4–5, 8% in the range of 5–6, and 13% in the range of 6–7. Yet, to be in line with the standard practice, 10 μM (pChEMBL value ca. 5.0) as a cut-off value of activity might have been more appropriate [83,84].

The hyperparameters of the sequential model of neural network in Keras/TensorFlow were optimized based on the prediction accuracy using the 5-fold cross-validation in order to assess modifications introduced to the model. The final sequential model consisted of an input layer followed by three hidden layers each containing 64 nodes with the Rectified Linear Unit (ReLU) activation function. The categorical cross-entropy loss function and the stochastic gradient descent optimizer with the softmax activation layer were applied.

The sequential neural network model was trained on the datasets that were randomly generated based on the curated ChEMBL data for chemokine receptors. For a final assessment, the original datasets were divided into training and testing sets in a 70/30 ratio and then used by NN. Five independent runs were used to compute the average (mean) results to minimize stochastic effects. There were two series of datasets—one with only active compounds and the other with active and inactive compounds included. The term ‘inactive compound’ means that this compound was present in the ChEMBL dataset for the certain receptor type, but its activity (measured as effective concentration, i.e., pChEMBL values) was less than 4.0 and it can be considered as an experimentally confirmed inactive compound. Thus, such a compound was treated as ‘inactive’ and represented a negative data point in the used training sets.

Results were evaluated by comparing known standardized activity values (pChEMBL) based on experimental results (functional assays) to the predicted values within each activity range. Results of NN were compared to results obtained with LightGBM. The latter algorithm was tested previously, but only for the CB1, CB2, GCGR, and GLP-1R receptor ligand datasets [56]. To perform an explicit comparison of these two approaches (NN and gradient boosted decision trees), a binary classification in NN was modified to fit a multi-class classification using ranges of activity values (pChEMBL ranges). Inactive compounds in each training set represented a set of negative data points. Including of this negative dataset representing true negatives was evaluated in terms of the accuracy of each predictor (NN vs. LightGBM and CCR1-6). The accuracy of the NN predictions was evaluated as a percentage of all predicted ligands that were classified as belonging to the correct activity class. In addition, ligands that were classified as belonging to a lower activity class (underpredicted) or to a higher activity class (overpredicted) were also reported.

For training LightGBM, a total training set for each receptor was used, one with only active compounds and the other which also included the inactive compounds. The number of leaves in the decision trees of LightGBM ranged from 7 to 200, the maximum depth varied from −1 to 10, the number of estimators from 50 to 500, and the learning rate ranged from 0.5 to 0.001, all with 5-fold cross-validation and R2 measure with grid tuning to the best estimator and the best parameters. The root mean square error (RMSE) between the experimental values (standardized pChEMBL values) and the predicted activity values was evaluated. To compare these results to the NN results, activity values predictions obtained from LightGBM were split into activity classes based on the same pChEMBL values, as described above for NN. NN and LightGBM results could then be directly compared despite their original discrete vs. continuous values, respectively.

### 2.4. Structure-Based Virtual Screening Assisted by NN and LightGBM

Compounds obtained in both structure- and ligand-based virtual screening for CCR2 and CCR3 were mapped against each other in order to find the compounds that overlapped between sets. In such way, 460 compounds proposed by SBVS for CCR2 and CCR3 could be limited to only a few compounds (10 and 12, respectively) that could be further tested in bioassays. NN predictions vs. SBVS predictions, LightGBM vs. SBVS, NN vs. LightGBM, and finally CCR2 vs. CCR3 datasets (for NN, LightGBM, and SBVS separately) were compared.

## 3. Results

### 3.1. Self-Docking to PDB Structures

The self-docking results are presented in Table 4, alongside information pertaining to PDB structures deposited in the PDB. All the CCR2 structures were in the inactive-state conformation. Based on the presented data, the PDB structure of CCR2 (6GPS) had the worst resolution in terms of structure-based virtual screening (SBVS). This impacted the results of the self-docking—though both 6GPS and 6GPX had the same ligand, the self-docking Glide scores and RMSD values were lower for 6GPX, suggesting that this was the better-quality structure. In the case of 5T1A despite the good resolution of the crystal structure, the Glide score value was similar to that of 6GPS and the RMSD was more than twice as high.

Most of the ligands present in the CCR5 structures were peptides. Such ligands demonstrate high flexibility and for the sake of computational time the standard precision (SP) Glide mode was used as the primary method for self- and cross-docking of them. The lowest RMSD values were obtained for structures with small-molecule ligands: 4MBS, 6AKX, and 6AKY; which means these ligands only slightly changed their orientations. These three structures were chosen to undergo more precise docking calculations due to the similarities in the structures of the ligands: compounds 21 and 34 were both derivatives of 1-heteroaryl-1,3-propanediamine derivatives [72] and designed to be alternatives of maraviroc, a drug used to treat HIV infection [85]. Regardless of which Glide mode (SP vs. XP) was used in self-docking, the lowest RMSD values were obtained for 6AKY (CCR5 with compound 34). Similarly, the best values of the Glide score were acquired for 4MBS—the receptor structure with maraviroc. For both of these structures, the results obtained using the SP and XP methods were remarkably similar; furthermore, the XP self-docking provided very similar values of the Glide score for both ligands, suggesting that the Glide-predicted affinity of compound 34 to CCR5 is comparable to that of maraviroc.

**Figure 1:**
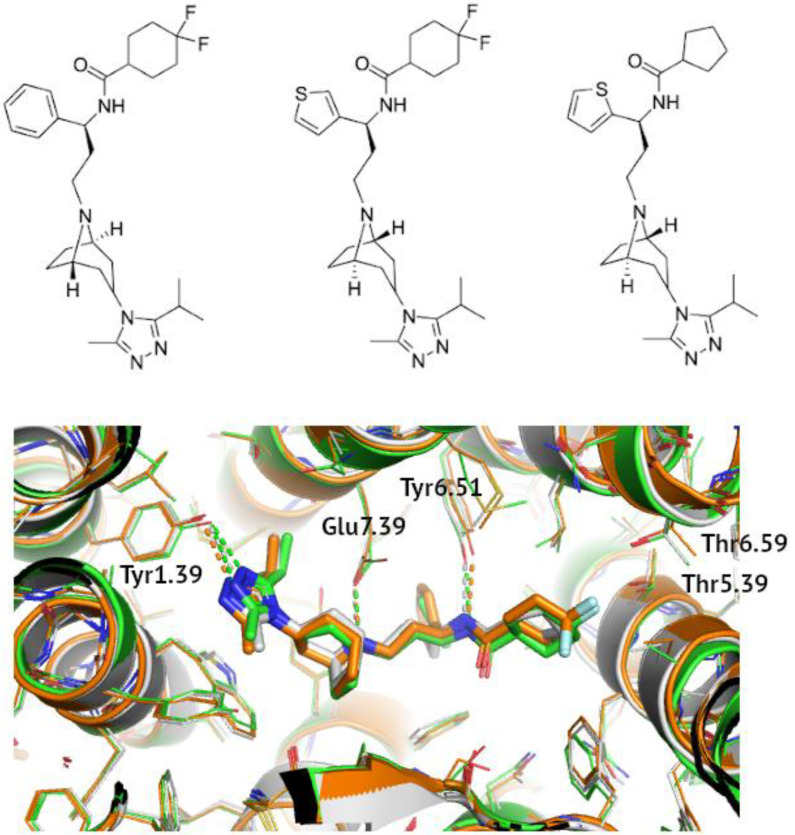
**A comparison of binding modes of known allosteric CCR5 inhibitors.** Maraviroc (4MBS, orange), compound 34 (6AKY, green), and compound 21 (6AKX, grey) were shown also in detail at the top from left to right. Polar contacts were marked with dashed lines and receptor residues were indicated in the Ballesteros-Weinstein notation.

The differences in values of the Glide score for these three ligands (maraviroc, compound 21, and 34) could perhaps be explained by taking a closer look at their structures, presented side by side in Figure 1. Compound 34 is the most similar to maraviroc, with only a phenyl ring having been changed to a tiophene. Both ligands possess a cyclohexane ring with two fluorine atoms. They are known to interact with the T195^5.39^ and T259^6.59^ residues of CCR5 [70]. The lack of these fluorine atoms in 6AKX with compound 21 and the subsequent lack of hydrogen bonds might explain higher values of the Glide score obtained for this structure. The phenyl group, on the other hand, is responsible for forming hydrophobic interactions with Y108^3.32^, F109^3.33^, F112^3.36^, W248^6.48^, and Y251^6.51^ [70]; in 6AKX and 6AKY, the thiophene ring plays a similar role in stabilizing the inactive conformation of the receptor. The positioning of the sulfur atom in the aromatic ring determined the depth to which it entered the binding pocket, with the sulfur atom in the *meta* position being buried deeper than that in the ortho position [72].

**Figure 2:**
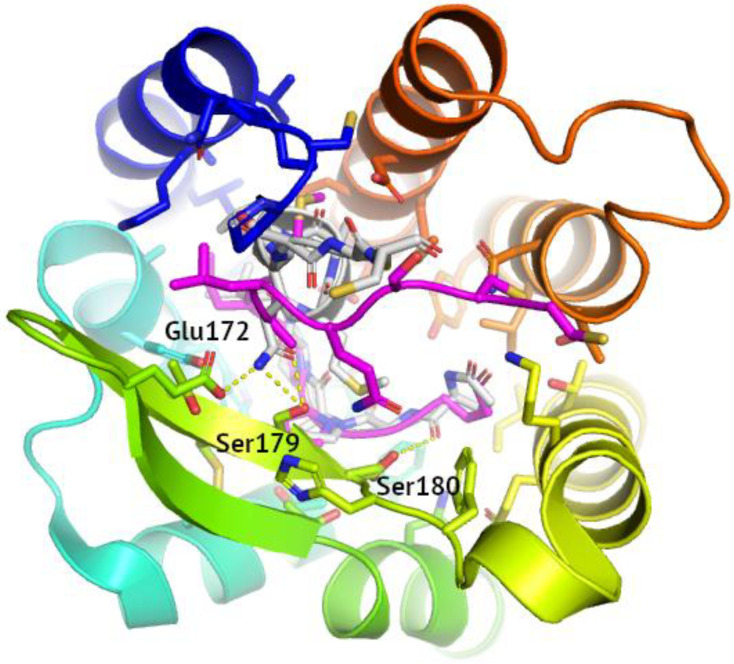
**Reconstruction of ligand binding mode in 5UIW self-docking.** The reference PDB pose of the 5UIW ligand was shown in grey, with the polar contacts involving side chains indicated with yellow dashed lines. The residues involved in polar contacts located in ECL2 were labeled. The Glide-reconstructed ligand pose was shown in magenta (RMSD equal to 4.19 Å).

The docking scores obtained for the structures containing peptide ligands were largely worse than in the case of the small molecules. The notable exceptions were 5UIW, where the docked ligand was CCL5, and 6MEO with the HIV-1 envelope spike. Of all the peptide ligand-containing structures, 5UIW had the best resolution and provided the lowest RMSD value in self-docking. The 5UIW ligand, truncated to the first 11 residues, was well-superposed on its PDB pose till residue Met5 (see Figure 2). However, a following helical turn visible in the PDB pose was not rebuilt by Glide. This justifies a 10-residue cutoff for docking of short peptides in Glide.

7F1T with only a slightly worse resolution, provided comparable results in terms of RMSD. Despite the low value of the Glide score, the RMSD value obtained for 6MEO was relatively high, suggesting the ligand had to change position regarding the original structure in order to improve the interactions with the receptor. All RMSD values obtained for peptide ligands were much larger than those obtained for small molecule ligands. This tendency can be also observed in the case of the CCR6 self-docking results.

### 3.2. Cross-Docking to PDB Structures

In the next step, ligands derived from crystal structures were subjected to cross-docking. The results are presented in Table 5. In the case of CCR2, the lowest value of the Glide score and lowest RMSD values were observed for the 6GPS ligand docked onto the 6GPX receptor, which agrees with the self-docking results. In turn, the ligand in the 6GPX structure demonstrated the best values of the Glide score when docked onto the 6GPS structure. When docked onto the 5T1A structure, however, both the 6GPS and 6GPX ligands displayed comparably high docking scores and RMSD values. The 5T1A ligand (BMS-681—orthosteric), however, demonstrated far better values of the Glide score and lower RMSD values when cross-docked onto the 6GPX structure, which, as was previously stated, had the best resolution. In conclusion, both the self- and cross-docking results suggest that of the three studied CCR2 structures, the 6GPX structure is of the highest quality in terms of structure-based virtual screening. In the case of the 4MBS, 6AKX, and 6AKY structures, cross-docking was performed using both SP and XP modes, like in the case of self-docking. As expected, the 4MBS ligand demonstrated the best scores when docked onto the 6AKY structure and vice versa, which can probably be attributed to the presence of the fluorine atoms and the hydrogen bonds they form (see above).

**Figure 3:**
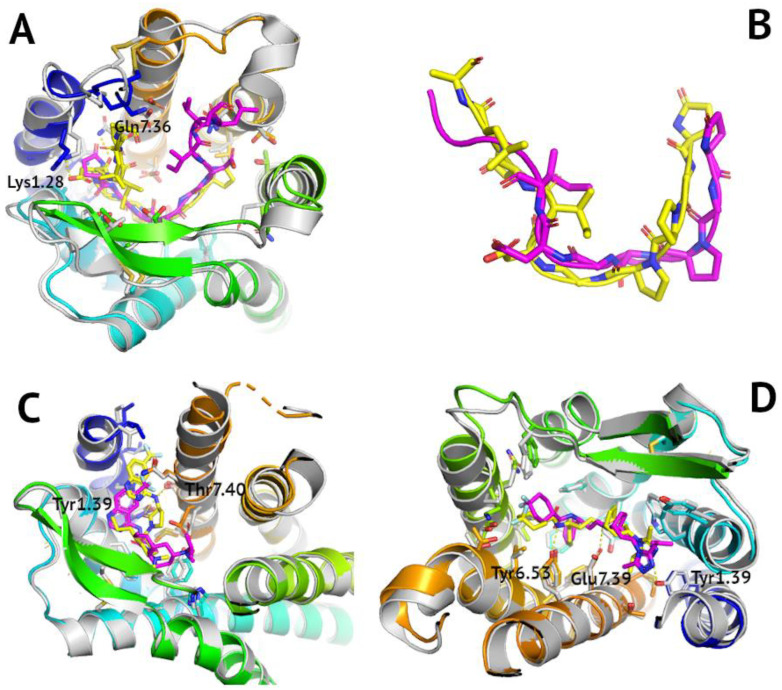
**Reconstruction of ligand binding modes in cross-docking of CC chemokine receptors.** The reference PDB poses, and respective PDB receptor structures were shown in grey. Polar contacts involving receptor residue side chains were shown as dashed yellow lines. Glide-generated ligand poses were shown in magenta, with receptors shown in blue-to-red. (**A**) cross-docking of the peptide, 10-residue long 7O7F ligand to 5UIW structure—RMSD equal to 3.69 Å, (**B**) overlay of 7O7F ligand conformations in PyMOL with RMSD equal to 2.68 (yellow—reference, magenta—reconstructed); (**C**) cross-docking of the small-molecule 5T1A ligand (an orthosteric one) to 6GPS structure—RMSD equal to 1.11 Å, (**D**) cross-docking of the small-molecule 4MBS ligand to 6AKX structure—RMSD equal to 1.1 Å.

Due to the large quantity of tested structures, SP cross-docking was performed using only the ligands from the 4MBS, 6MEO, and 7O7F. The 4MBS ligand demonstrated the worst results (the highest values of the Glide score and largest RMSD values) when docked onto the 7F1Q, 7F1R, and 7F1S structures. This was likely since the 4MBS ligand as an inhibitor favored structures including the inactive-state receptor and not structures with the active-state receptor (7F1Q, 7F1R, and 7F1S). Similar results were expected for the 6MEO ligand as it was also an inhibitor. Indeed, this ligand demonstrated the best results when cross-docked onto the inactive receptor structures (4MBS, 6AKX, 5UIW). However, although the 6MEO ligand demonstrated the worst results of the three active structures (7F1Q, 7F1S, 7O7F), values of the Glide score were also relatively high for the inactive-state structures (6MET, 4AKY). This was because the 6MEO ligand was a peptide and not a small molecule such as the 4MBS ligand. Thus, it demonstrated better fitness to the peptide-bound receptor conformations and not necessarily the inactive-state receptor conformations. The 7O7F ligand (agonist), contrary to what was expected, displayed larger RMSD values when docked onto active-state receptor structures (particularly 7F1S). However, there was not as much of a disparity between the different docking scores as in the case of the previous ligands to assess if the 7O7F ligand indeed favored the active receptor conformations. What is more, molecular docking of peptide compounds, such as the 7O7F ligand, requires a more extensive conformational search than in case of small-molecule ligands to account for their conformational variability. However, because of the limited computational time, the SP mode was used for these peptide compounds instead of XP. The lowest RMSD value (3.69) for the 7O7F ligand was obtained for the 5UIW structure; however, the ligand was flipped inside the binding site (see Figure 3A). Nevertheless, the reconstruction of its peptide, 10-residue long conformation was proper—with RMSD equal to 2.68 Å (computed in PyMOL, see Figure 3B). This again confirms that the 10-residue cutoff is the best for the peptide docking in Glide.

### 3.3. ROC Analysis of CC Chemokine Receptor Models

ROC curves and enrichment factors provide important information on the protein model ability to distinguish actives from inactive ligands in datasets [58]. They represent an accurate and simple method of distinguishing good protein models from bad models in terms of SBVS, as shown by us on the example of glucagon receptors [58,86]. Since the ligands obtained from ChEMBL were mostly inhibitors, it was assumed that the created ROC curves would be better for the receptors in their inactive state. Models used in the actives enrichment analysis are presented in Supplementary Table S3 and Figure S1.

For CCR1, the above hypothesis was indeed true, but only in the case of the Robetta-generated model (see Supplementary Table S4). The inactive-state receptor model generated by Robetta indeed demonstrated the best ROC curve, signified by the larger area under the curve (AUC). However, the GPCRdb inactive-state model recognized actives from decoys, but this classification started to improve in the middle of the ROC curve. This means that for smaller datasets this receptor model tended to misclassify actives from inactive ligands (high false positives rate). Superposition of these two inactive-state CCR1 models in PyMOL showed that TM helices were well-aligned (see Supplementary Figure S1). However, there were significant differences between these two models in the conformations of intra- and extracellular fragments. The largest differences were visible between helix H8, the C- and N-termini, as well as loops ICL1, ICL3, and ECL1. The most significant difference was in the N-termini. It was longer in the GPCRdb model than in the Robetta model and resembled the active-state conformation of the N-termini in other solved structures of CC chemokine receptors. Most probably, additional interactions of ligands with the extended N-termini were the reasons why the GPCRdb model tended to overpredict actives (high false positives rate) and displayed an S-shaped ROC curve. This again [58,86] confirms that ROC curves are successful in the assessment of the quality of GPCR models.

The active ligands enrichment for CCR2 was much better than those for CCR1 (see Supplementary Table S4). As expected, the active-state receptor models performed the worst, but the intermediate-state receptor model obtained from GPCRdb was shown to accurately distinguish between the ChEMBL-derived actives (inhibitors of the CCR2 mediated signaling) and DUD-E decoys. It suggests that it could be used in further SBVS studies alongside the inactive-state PDB structure of CCR2 (e.g., 6GPX).

**Figure 4:**
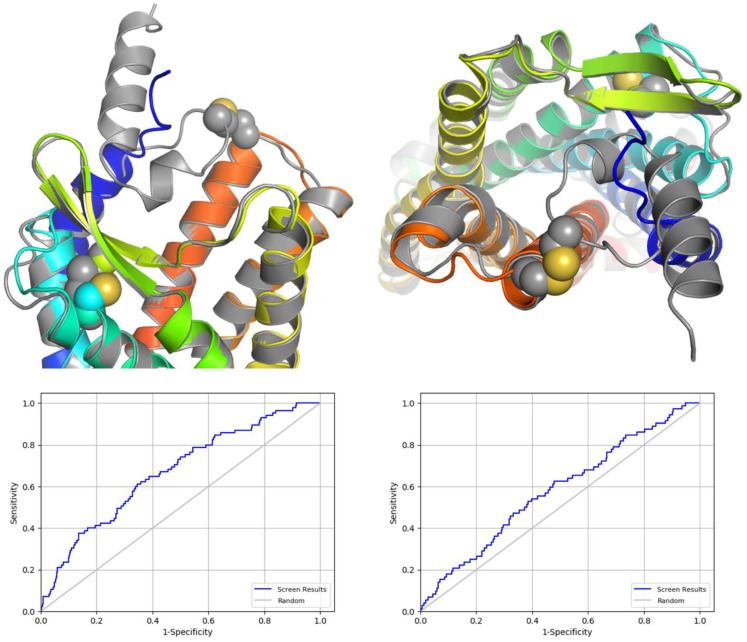
A comparison of the inactive CCR3 models generated with Robetta (grey), and deposited in GPCRdb (blue-to-red). The disulfide bridges were marked as spheres. Below—ROC curves for these two models of inactive-state CCR3, for Robetta model (**left**) and for GPCRdb model (**right**).

The CCR3 models also performed well, with the best ROC curves being obtained for the active-state model deposited in GPCRdb and inactive-state model generated with Robetta (see Figure 4). Yet, active-state models (GPCRdb and I-TASSER) also performed well in the actives enrichment study. The N-terminus in the inactive-state GPCRdb model of CCR3 was similar to N-terminal fragments which interacted with chemokines in 7F1Q, 7F1T, and 7O7F active-state PDB structures of CCR5. Namely, it was moved away from the orthosteric binding site towards ECL2. In contrast, the N-terminus in the inactive-state Robetta model of CCR3 was much closer to the orthosteric binding site, partly substituting a chemokine and partly forming a cap on the extracellular part of the receptor. Importantly, N-terminus and the beginning of TM1 in this inactive-state Robetta model was very much like the PDB structure of CCR5 (4MBS), indeed representing the inactive state of the receptor. This could be the reason why the Robetta model outperformed the GPCRdb model in distinguishing CCR3 actives from inactive ligands as observed in ROC curves (see Figure 4). In addition, the Robetta model contained a second disulfide bridge in the extracellular part of the receptor (ECL3—N-terminus) besides the typical GPCR disulfide bridge joining ECL2 and TM3. This additional disulfide bridge was present in most CC chemokine receptor PDB structures but was not present in the inactive-state GPCRdb model of CCR3.

The ROC curves obtained for the active and inactive-state CCR4 models were like a random classifier, which means that they hardly could be used efficiently in SBVS. However, the inactive-state Robetta model was again the best and again included N-termini forming the receptor cap which substituted a chemokine, joined with ECL3 by the disulfide bridge. I-TASSER and GPCRdb models again included N-termini resembling the active-state receptor conformations. CCR5 was not tested in the active ligands enrichment as there were already many active and inactive-state structures of this receptor in PDB. The CCR6 models from GPCRdb also demonstrated random classifier-like ROC curves, though the best one was for the intermediate-state receptor model. The described above ROC curves were presented in Supplementary Table S4.

Robetta used the inactive-state 4MBS structure as a template for all described models and most probably it was the reason why Robetta models performed the best in discriminating decoys from inhibitors, as demonstrated by ROC curves. In the 4MBS structure, the N-terminus was directed towards the receptor rather than away from it, which would explain why this region in all Robetta models had a similar conformation. Furthermore, there was a disulfide bond between the N-terminus and ECL3 in both the 4MBS template and the CCR3 and CCR4 Robetta models. It would also likely have been present in the CCR1 Robetta model if the N-terminus had not been truncated.

In addition to the overall model quality by the ROC curves analysis, SiteMap [87] was used to determine the location of the binding sites in the CCR2 Robetta model, which was then compared to the 4MBS CCR5 structure. The 4MBS ligand fits into the predicted binding sites, lending credence the quality of the model. This comparison is presented in Supplementary Figure S2.

### 3.4. Structure-Based Virtual Screening Involving CCR2/CCR3 Receptor Subtype Selectivity

In both cases, CCR2 and CCR3, 460 different ligands extracted automatically in Maestro as the best-scoring fraction of the results obtained from Glide were divided into 22 different clusters. The number of ligands assigned to each cluster, as well as information about the ligand closest to the centroid (medoid), can be found in Supplementary Table S5 (for CCR2) and Table S6 (for CCR3). All medoid ligands could be classified as actives based on values XP Gscore (below −8). For CCR2, the best XP Gscore value was obtained for the ligand belonging to cluster 11; for CCR3, it was the ligand belonging to cluster 8. The residues that were involved in ligand binding and numbered according to the Ballesteros-Weinstein notation (see Supplementary Figure S3) were presented in Supplementary Figures S4 and S5.

As for the receptor subtype selectivity, CCR3 ligands occupied the center of the receptor, while CCR2 ligands (incl. the 6GPX ligand) were slightly moved to the right, to TM1 and TM7 (see Supplementary Figures S4 and S5). In both, CCR2 and CCR3, Glu7.39 and Tyr6.51 were involved in ligand interactions, while Tyr1.39 only in CCR2. Residue 4.60 was involved in ligand interactions in both receptors, as Asn in CCR2 and Glu in CCR3. Arg1.28 in CCR3 were involved in interactions with VS-extracted ligands but not in 6GPX and to much less extent in any of VS-extracted CCR2 ligands (as Lys1.28).

### 3.5. Ligand-Based Drug Design Involving Machine Learning

**Figure 5:**
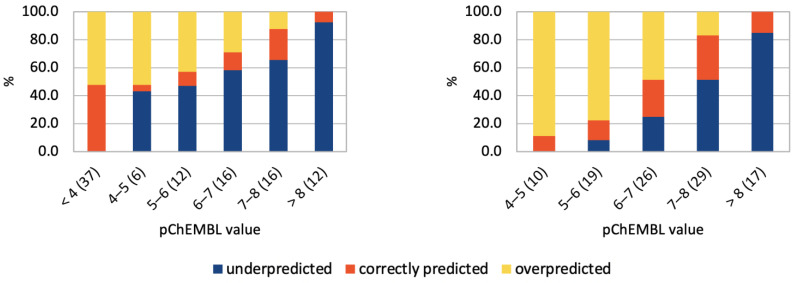
**Compound activity predictions for CCR2 using the sequential Keras/TensorFlow model of NN.** Histogram of ligand activities as predicted by Keras/TensorFlow. Ligand activities (pChEMBL) were divided into ranges (x-axis). The fraction of the dataset that was assigned to each activity range (in %) was given in brackets. Predicted activity values fell into three categories: overpredicted, underpredicted, and predicted correctly, with (**left**) and without (**right**) inactive compounds included in the datasets.

The overall accuracy for the neural network prediction of the ligand activity to the set of six chemokine receptors was equal to 40% with ‘non-active’ compounds included but dropped to 23% when ‘inactives’ were removed. The overall average prediction accuracy ranged from 20% (CCR1) to as high as 86% (CCR6) (see Figure 5 and Supplementary Table S7). However, a detailed analysis of the data revealed that there were clear prediction biases generated from discrepancies within the distribution of the known experimental data. For example, in CCR1, where the actives dataset was biased towards activities in the high nanomolar range, the best predictions were for compounds of this activity range. This suggests that the prediction model could be overfitted to the training data set. This is even more clearly illustrated by datasets for the five other receptors, in which the proportion of inactive compounds was also biased. This overfitting of the model was most clearly observable for CCR6, in the dataset of which 88% of the ligands in each trial, on average, had activity between 10 and 100 µM. Compounds in this activity range were correctly classified in 98% of cases, while inactive compounds were overpredicted in 100% of cases, and compounds of higher activity were underpredicted in 100% of cases. For CCR2, the dataset of which included 37% inactive compounds, the NN performed the best in classification of this type of compounds while underpredicted the activities of all actives. For CCR4, the dataset included 64% of inactive compounds. The NN classified these compounds correctly in 83.1% of cases and underpredicted activities of other compounds. On the other hand, for CCR3, which dataset was biased towards the high activity compounds (78% of compounds had activities better than 1 µM) there was a tendency to overpredict activity. Compounds with activity worse than 10 µM were overpredicted in 91% of cases, though they constituted only 7% of the dataset on average. For CCR5, which had a bimodal activity distribution (32% ‘inactives’, 41% of better activity than 100 nM), relatively few compounds were correctly classified, but rates of under- and overprediction were usually equivalent.

Repeating training and testing of the NN with datasets in which inactive compounds (the negative dataset) were removed enhanced the described above trends. Activity values in datasets were much more evenly distributed, but unexpectedly this led to worse overall performance of the model. The average prediction accuracy was 21% (CCR4) to almost 26% (CCR5). Notably, the medium-activity compounds were the most accurately predicted, while the low-activity compounds were overpredicted and the high-activity compounds were underpredicted, which suggests a tendency of the NN to overfitting. It was most evident for CCR5, in which dataset the previous bimodal distribution of activity values (with one mode centered on ‘inactives’) was removed, leading to 34% average accuracy for compounds with activities better than 100 nM (60% of the dataset on average). The CCR6 dataset could not be examined this way because it almost did not include any inactive compounds.

**Figure 6:**
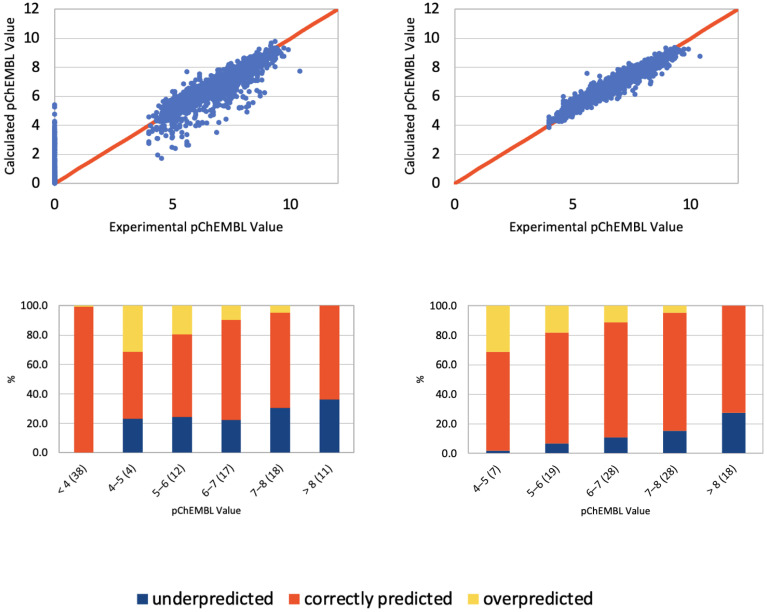
**Compound activity predictions for CCR2 using LightGBM.** (**Top**): Comparison of compound activities predicted by LightGBM compared to the known activity values (as defined by pChEMBL values), with (**left**) and without (**right**) inactive compounds included in the datasets. A perfect correlation line is included for comparison (red line). (**Bottom**): Histograms representing LightGBM result in prediction of activity values. Ligand activities (pChEMBL) were divided into ranges (x-axis). The fraction of the dataset that was assigned to each activity range (in %) is included in brackets. Predicted activity values fell into three categories: overpredicted, underpredicted, and predicted correctly. (**Left**)—with inactive compounds included, (**right**)—without ‘inactives’ included.

The results described above clearly indicate that the NN used here was prone to overfitting. Therefore, the preparation of uniformly distributed training datasets is important to avoid any bias in the model predictions. Overall, gradient boosting decision trees (LightGBM) provided qualitatively better predictions for used training sets (see Figure 6 and Supplementary Tables S8 and S9) as this algorithm is more fitted to numerical values predictions (pChEMBL values) instead of binary classification (active/inactive), for which NNs are typically used. For example, LightGBM assigned the proper activity value range for compounds in 84.9% of cases on average for CCR5 datasets without inactive compounds. The average performance of LightGBM ranged from 52.9% (CCR1 with ‘inactives’ included) to the aforementioned results for the CCR5 dataset.

### 3.6. Structure-Based Virtual Screening Assisted by NN and GBM

**Figure 7:**
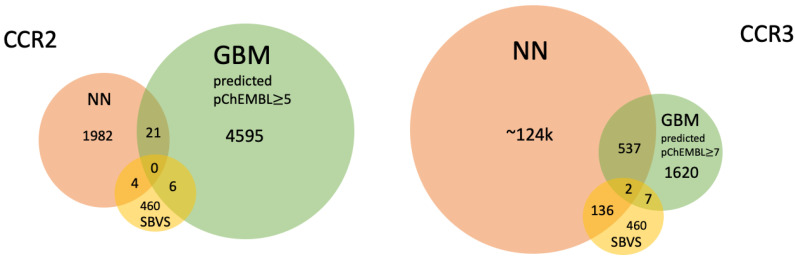
Venn diagrams presenting the overlap between the compounds found through SBVS, NN, and GBM. The diagram on the **left** presents the results for the 6GPX structure of CCR2, and on the **right** for the inactive-state Robetta model of CCR3.

**Figure 8:**
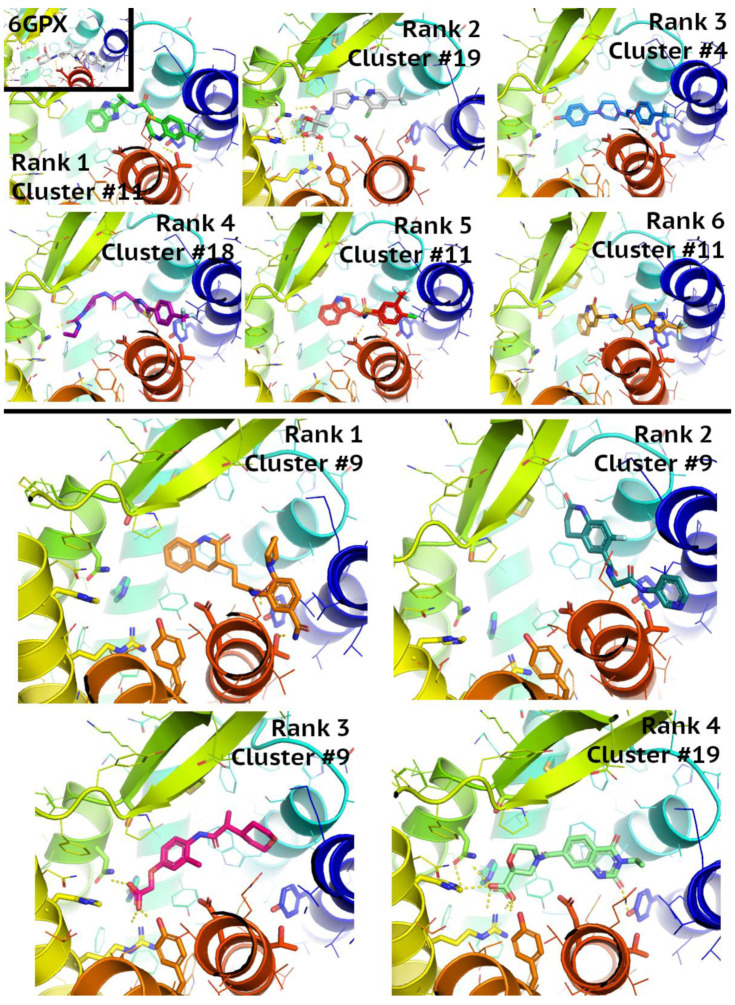
Interactions between the best-ranked ligands found through virtual screening using the CCR2 6GPX structure. The top displays the ligands that overlapped in both the GBM and SBVS results, and the bottom those that overlapped in both the NN and SBVS results.

**Figure 9:**
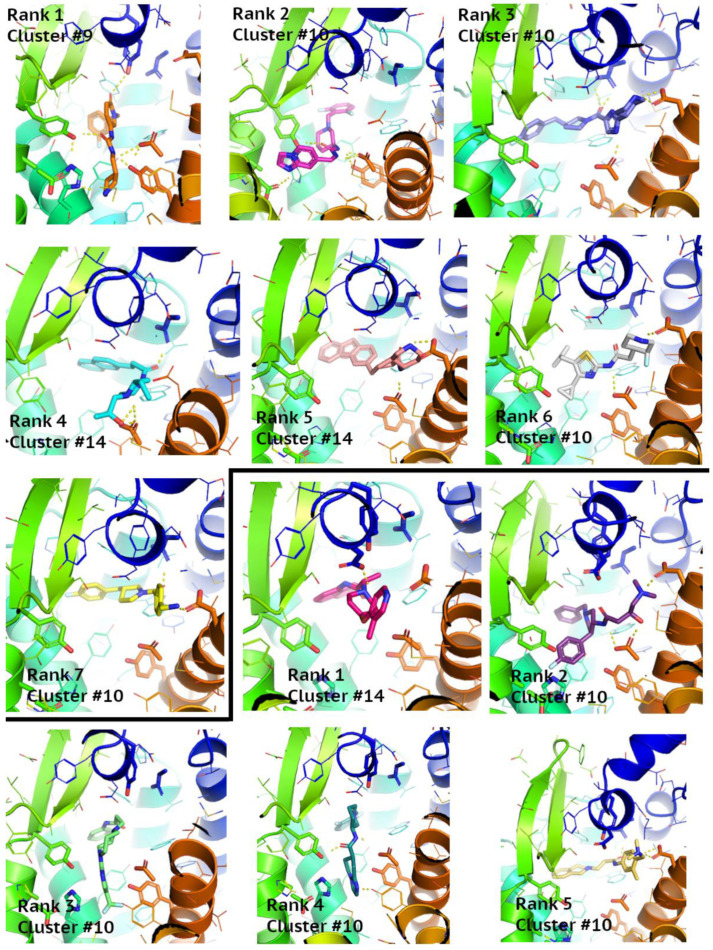
Interactions between the best-ranked ligands found through virtual screening and the inactive-state CCR3 Robetta model. The top displays the ligands that overlapped in both the GBM and SBVS results, and the bottom those that overlapped in both the NN and SBVS results. Compounds ranked as 2nd and 5th by GBM were also among compounds predicted by NN.

The overlap between the Enamine compounds selected by NN, GBM, and SBVS is shown on the Venn diagrams in Figure 7. It provided 10 new active compounds for CCR2 and 12 for CCR3 (see Figure 8 and Figure 9, and Supplementary Tables S10–S13). For CCR2, the largest number of compounds was supplied by GBM (over 4000 compounds), and while there was some overlap, there was not a single compound that had been selected by all three methods. For the CCR3 model, however, two such compounds were discovered, suggesting that they would present a good basis for further research. The interactions between the best-ranked ligands and the appropriate receptors were shown on Figure 8 (CCR2) and Figure 9 (CCR3). Here, the compounds were ranked according to XP-Gscore values obtained from Glide. In the case of the NN-predicted actives of CCR3, only five top-scoring (according to XP-Gscore) compounds were presented in Figure 9. More information about these ligands, including their interactions with their receptor, is provided in Supplementary Tables S10–S13.

A total of 537 CCR3 active compounds predicted by both NN and GBM were in the range of pChEMBL values 7.0–8.3, while 21 CCR2 active compounds were in the range of pChEMBL values 5.0–6.7. The two CCR3 active compounds predicted by these three methods were Z1426245621 and Z2441027668 with GBM-predicted pChEMBL values of 7.23 and 7.12, respectively. XPGscore values for these two compounds were below −10 (see Supplementary Table S12). Interestingly, both compounds belonged to highly populated clusters (10th and 14th with 264 and 39 similar compounds, respectively). The binding modes of these compounds were presented in Figure 9—compounds ranked as 2nd and 5th among GBM-predicted actives. The compound ranked as 2nd occupied the center of the receptor, while the compound ranked as 5th was moved closer to TM1 and TM7, such as an CCR2 antagonist in the 6GPX structure (see Figure 8, left upper corner). Both compounds formed polar contacts with Glu7.39, which is also involved in antagonist-receptor interactions in 6GPX (see Supplementary Figure S4).

Subsets of CCR2 and CCR3 active ligands predicted by LightGBM mostly did not overlap. Only 30 compounds in the predicted CCR2 subset overlapped with the other receptor subset. The overlap between subsets generated for different receptors with different ML methods was even smaller. Only four compounds in the NN-generated CCR2 subset were also in the predicted CCR3 subset generated with LightGBM. In the case of the NN-based classifier, the overlap between CCR2 and CCR3 predicted active ligands was larger. Namely, 507 ligands in the CCR2 subset were also in the predicted CCR3 subset. It was mostly due to a large population of active ligands predicted as belonging to the category of the highest activity (~124k ligands, see Figure 7). This suggests an important conclusion that the LightGBM-based model reflects the receptor subtype selectivity during prediction. On the other hand, the NN model is a much weaker predictor, with a high fraction of false positives among predictions. This confirms the overall performance of the NN model, as described above (see Figure 5). SBVS performed equally well as LightGBM in distinguishing the receptor subtype. Only 8 compounds out of 460 were found to be in both the CCR2 and CCR3 subsets of predicted active compounds. Interestingly, none of these CCR2/CCR3 non-selective eight compounds were found in either the NN or LightGBM predicted actives.

## 4. Discussion and Conclusions

Chemokine receptors represent promising drug targets regarding numerous diseases, such as infections, allergies, and inflammation. Information on how chemokine receptors evolved and what impact they have on a variety of cellular processes is important for accurate target selection during drug discovery. Besides the selection of a drug target, another difficulty is a limited access to the structures of all the activation states of chemokine receptors due to experimental difficulties regarding GPCR structure determination. This significantly hinders structure-based drug discovery. Publicly available models of chemokine receptors deposited in GPCRdb or generated with GPCRM, Robetta, I-TASSER or other web services are valuable sources of structural information provided the model quality can be properly assessed in advance. This study aimed to determine the differences between various PDB structures that could be relevant in structure-based VS. In the case of homology models, we aimed at proposing a simple but effective approach to assess the model quality in prior to SBVS. This approach had already been tested before for class B GPCR receptors (GCGR, GLP-1R, VPAC1, VPAC2, PAC1) and now it has been tested for class A chemokine receptors. From among the available CCR2 structures, 6GPX was suggested to be the best for SBVS for small-molecule active ligands, while evaluating different CCR5 structures proved to be more complicated due to the presence of peptide ligands in these structures. Following the enrichment and molecular docking study, SBVS was performed using 6GPX against the Enamine screening library HLL-460. As a result, new active compounds of CCR2 were proposed. These compounds, to our knowledge, were not tested before in bioassays including this receptor. In the same manner, the CCR3 inactive-state Robetta model was used to propose new active ligands of this receptor.

In addition to SBVS, ChEMBL datasets for CCR1–6 were used as training sets for two machine learning algorithms (Keras/TensorFlow sequential NN and LightGBM). The performance of these two algorithms in ligand-based drug design for CC chemokine receptors was compared. LightGBM outperformed NN regardless of whether the negative datasets (ligands that were confirmed to be inactive in functional assays—‘inactives’) were included or not.

The NN model—a multi-class classifier based on Keras/TensorFlow—split the tested compounds into six classes corresponding to the six ranges of pChEMBL values. These predictions, however, were susceptible to the same biases that were present in the training data. For example, 78% of the training dataset for CCR3 (with ‘inactives’ included) constituted active compounds with pChEMBL ≥ 6.0 while inactive compounds (pChEMBL < 4.0) constituted only 1%. Consequently, 124,967 compounds (27% of the Enamine dataset) were classified as actives (6th class). Conversely, the CCR2 training dataset (with ‘inactives’ included) was biased towards inactive compounds—37% with 28% of compounds in the two highest activity classes. This training set was more evenly distributed between active and inactive compounds than in case of CCR3 for which 58% of compounds were in the two highest activity classes but only 1% in the inactive compounds class. To further improve the activity prediction alterations in the ChEMBL original datasets would have to be done to balance the active/inactive compound shares in the training sets. Nevertheless, adding the negative dataset (true negatives, ‘inactives’) improved the binary classification (active vs. inactive) regardless of whether NN or LightGBM was used.

For the GBM model to perform equally well for CCR2 and CCR3 (<5000 and <2000 active compounds in the Enamine dataset, respectively) the activity cutoff would have to be changed from 5.0 to 7.0 for CCR2 and CCR3, respectively. Without changing this cutoff, the GBM model predicted 216,407 compounds as CCR3 actives which is even more than 124k compounds predicted as CCR3 actives by the NN model. Thus, the superiority of the GBM model over the NN model is mainly because it provided continuous activity values in contrast to the discrete activity ranges of the NN model. With such continuous activity values, it is easy to extract, e.g., top 1000 compounds for further investigations which is not possible with only six activity ranges used for the NN model. In such cases, increasing the number of classes for the NN classifier could solve this problem.

Despite the above limitations of the GBM and NN models they proved to be useful when combined with SBVS. NN and GBM-predicted datasets of CCR2 and CCR3 actives were juxtaposed with datasets of actives obtained in SBVS. The ML and SBVS-generated datasets overlapped in a few cases which allowed 10 new active CCR2 ligands and 12 new active CCR3 ligands from the Enamine compound library to be proposed. Among the predicted CCR3 ligands, there were two compounds predicted as actives by all three methods: NN, GBM, and SBVS. These two compounds constitute the basis for further investigations regarding small-molecule inhibitors of the CCR3 signaling. This combined approach including both structure-based virtual screening and ligand-based drug design based on machine learning proved as a simple, low-cost, and effective method for CC chemokine receptors drug discovery.

## Figures and Tables

**Table 1:** CC chemokine receptors and their ligands [9,10].

CC Chemokine Receptor	Chemokines	Immune Cell Subset	Disease
CCR1 (CD191)	CCL3, CCL5 to CCL8, CCL13 to CCL16, and CCL23	peripheral blood lymphocytes monocytes	MS ^1^, transplant, RA ^2^, asthma, nephritis [11]
CCR2 (CD192)	CCL2, CCL7, CCL8, CCL11, CCL13, and CCL16	monocytes activated memory T cells, B cells, and basophils (in humans) in peritoneal macrophages (in mice)	MS, RA, transplant, asthma, atherosclerosis [11]
CCR3 (CD193)	CCL2, CCL5, CCL7, CCL8, CCL11, CCL13, CCL15, CCL18, CCL24, CCL26, and CCL28	eosinophils, basophils, and Th1 and Th2 cells allergic reactions	asthma, allergies [12]
CCR4 (CD194)	CCL3, CCL5, CCL17, and CCL22	Th2 T lymphocytes T cell receptor activation and trafficking of dendritic cells	asthma [13]
CCR5 (CD195)	CCL2 to CCL5, CCL8, CCL11, CCL13, CCL14, and CCL16	peripheral blood-derived dendritic cells CD34+ hematopoietic progenitor cells in certain activated/memory Th1 lymphocytes	MS, transplant, RA, asthma, nephritis, IBD ^3^, AIDS ^4^ [11]
CCR6 (CD196)	CCL20	inactivated memory T-cells Th17 cells some dendritic cells	SLE ^5^ [14]

^1^ multiple sclerosis, ^2^ rheumatoid arthritis, ^3^ inflammatory bowel disease, ^4^ acquired immunodeficiency syndrome, ^5^ systemic lupus erythematosus.

**Table 2:** CC chemokines expressed during viral infections with respective chemokine receptors binding them.

CC Chemokine	Viruses inducing the Chemokine Expression	Chemokine Receptors
CCL1 [26,27]	MHV-68 (murine Herpes virus-68) RSV (respiratory syncytial virus)	CCR8
CCL2 [27,28,29]	DENV (Dengue virus) HSV (Herpes simplex virus) Influenza virus PVM (Pneumonia virus of mice) RSV (respiratory syncytial virus) MHV (mouse hepatitis virus) TMEV (Theiler’s murine encephalomyelitis virus) pMuLV (polytropic murine leukemia viruses) VSV (vesicular stomatitis virus) LCMV (lymphocytic choriomeningitis virus)	CCR2, CCR4
CCL3 [27,30,31,32,33]	DENV (Dengue virus) HSV (Herpes simplex virus) Influenza virus MCMV, murine cytomegalovirus MHV-68 (murine Herpes virus-68) PVM (Pneumonia virus of mice) RSV (respiratory syncytial virus) MHV (mouse hepatitis virus) pMuLV (polytropic murine leukemia viruses) VSV (vesicular stomatitis virus) LCMV (lymphocytic choriomeningitis virus) HIV (Human Immunodeficiency Virus)	CCR4, CCR5
CCL4 [27,30,32,34,35]	DENV (Dengue virus) HSV (Herpes simplex virus) RSV (respiratory syncytial virus) MHV (mouse hepatitis virus) pMuLV (polytropic murine leukemia viruses) TMEV (Theiler’s murine encephalomyelitis virus) VSV (vesicular stomatitis virus) LCMV (lymphocytic choriomeningitis virus) HIV (Human Immunodeficiency Virus)	CCR1, CCL3, CCR5, CCR8
CCL5 [27,32,36]	HSV (Herpes simplex virus) Influenza virus MHV-68 (murine Herpes virus-68) HCV (hepatitis C virus) PVM (Pneumonia virus of mice) RSV (respiratory syncytial virus) MHV (mouse hepatitis virus) pMuLV (polytropic murine leukemia viruses) TMEV (Theiler’s murine encephalomyelitis virus) VSV (vesicular stomatitis virus) LCMV (lymphocytic choriomeningitis virus) HIV (Human Immunodeficiency Virus) DENV (dengue virus)	CCR1, CCR3, CCR4, CCR5
CCL6 [27,37]	TMEV (Theiler’s murine encephalomyelitis virus)	CCR1
CCL7 [27,38,39]	HSV (Herpes simplex virus) PVM (Pneumonia virus of mice)	CCR1, CCR2, CCR3, CCR5, CCR10
CCL8 [24,27,40,41]	HSV (Herpes simplex virus)	CCR1, CCR2, CCR3, CCR5
CCL11 [27,42,43]	PVM (Pneumonia virus of mice)	CCR2, CCR3, CCR5

**Table 3:** Available crystal structures and models of CC chemokine receptors.

Receptor	Conformational State	PDB ID	Method
CCR1	active	—	GPCRdb, I-TASSER
	intermediate	—	I-TASSER
	inactive	—	GPCRdb, Robetta
CCR2	active	—	GPCRdb
	intermediate	—	GPCRdb
	inactive	5T1A [66], 6GPS [67], 6GPX [67]	—
CCR3	active	—	GPCRdb, I-TASSER
	inactive	—	GPCRdb, Robetta
CCR4	active	—	GPCRdb, I-TASSER
	inactive	—	GPCRdb, Robetta
CCR5	active	7F1Q [68], 7F1R [68], 7F1S [68], 7O7F [69]	—
	inactive	4MBS [70], 5UIW [71], 6AKX [72], 6AKY [72], 6MEO [73], 6MET [73], 7F1T [70]	—
CCR6	active	6WWZ [74]	—
	intermediate	—	GPCRdb
	inactive	—	GPCRdb

**Table 4:** Self-docking results.

Receptor	PDB ID	Resolution [Å]	Ligand	Small-Molecule Ligand	Glide Score	RMSD ^1^
CCR2	5T1A	2.81	BMS-681	yes	−7.777 ^2^	0.96
	6GPS	3.30	MK-0812	yes	−7.933 ^2^	0.46
	6GPX	2.70	MK-0812	yes	−9.103 ^2^	0.25
CCR5	4MBS	2.71	maraviroc	yes	−9.026 ^3^	0.78
					−9.004 ^2^	0.74
	5UIW	2.20	5P7-CCL5	no	−9.494 ^3^	4.19
	6AKX	2.80	compound 21	yes	−7.102 ^3^	0.55
					−7.79 ^2^	1.19
	6AKY	2.80	compound 34	yes	−8.961 ^3^	0.36
					−9.002 ^2^	0.29
	6MEO	3.90	HIV-1 envelope spike	no	−9.433 ^3^	10.13
	6MET	4.50	HIV-1 envelope spike	no	−5.863 ^3^	5.22
	7F1Q	2.90	MIP-1α and G_i_	no	−4.898 ^3^	7.70
	7F1R	3.00	RANTES and G_i_	no	−6.900 ^3^	7.83
	7F1S	2.80	apo receptor in complex with G_i_	no	—	—
	7F1T	2.60	MIP-1α	no	−7.149 ^3^	4.36
	7O7F	3.15	[6P4]CCL5	no	−7.875 ^3^	7.34
CCR6	6WWZ	3.34	CCL20	no	−9.275 ^3^	6.78

^1^ computed for heavy atoms with respect to the PDB structure, ^2^ computed using the extra precision (XP) mode, ^3^ computed using the standard precision (SP) mode.

**Table 5:** The cross-docking results.

Receptor	Ligand	Structure	Glide Score	RMSD ^2^
CCR2	5T1A	**6GPS ^1^**	**−8.281 ^3^**	**1.11**
		6GPX	−5.383 ^3^	4.39
	6GPS	**6GPX**	**−9.028 ^3^**	**0.39**
		5T1A	−3.940 ^3^	9.94
	6GPX	**6GPS**	**−8.378 ^3^**	**0.69**
		5T1A	−4.919 ^3^	10.00
CCR5	4MBS	**6AKX**	**−8.834 ^4^**	**0.54**
			**−8.646 ^3^**	**1.1**
		**6AKY**	**−7.980 ^4^**	**0.74**
			**−8.912 ^3^**	**0.61**
		6MET	−5.214 ^4^	2.25
		6MEO	−6.025 ^4^	2.51
		5UIW	−5.940 ^4^	3.98
		7O7F	−5.464 ^4^	4.53
		7F1T	−5.126 ^4^	4.55
		7F1Q	−2.975 ^4^	4.73
		7F1R	−3.754 ^4^	5.78
		7F1S	−3.735 ^4^	6.73
	6MEO	**6AKX**	**−11.31 ^4^**	**7.89**
		**6AKY**	**−7.833 ^4^**	**8.33**
		**4MBS**	**−12.929 ^4^**	**8.95**
		7F1Q	−8.798 ^4^	9.36
		6MET	−7.165 ^4^	9.74
		5UIW	−10.527 ^4^	10.12
		7F1R	−6.731 ^4^	10.45
		7F1S	−7.995 ^4^	10.67
		7O7F	−7.963 ^4^	10.84
		7F1T	−6.738 ^4^	12.23
	7O7F	**5UIW**	**−6.550 ^4^**	**3.69**
		**6MEO**	**−7.851 ^4^**	**5.14**
		**4MBS**	**−7.643 ^4^**	**5.63**
		6MET	−6.414 ^4^	6.26
		6AKY	−7.268 ^4^	7.09
		6AKX	−7.223 ^4^	7.18
		7F1Q	−6.037 ^4^	7.48
		7F1T	−7.108 ^4^	8.29
		7F1R	−6.983 ^4^	8.79
		7F1S	−7.329 ^4^	13.03
	6AKX	4MBS	−8.501 ^4^	0.89
		**6AKY**	**−8.325 ^4^**	**0.49**
	6AKY	**4MBS**	**−8.716 ^4^**	**0.73**
		6AKX	−8.282 ^4^	1.04

^1^ results with the lowest values of RMSD were bolded, ^2^ computed for heavy atoms with respect to the crystal structure, ^3^ computed using the extra precision (XP) mode, ^4^ computed using the standard precision (SP) mode.

## Data Availability

All relevant data was included within the article or supplementary materials.

## Supplementary Materials

The following supporting information can be downloaded at: https://www.mdpi.com/article/10.3390/pharmaceutics15020516/s1, Figure S1: A comparison of the inactive-state CCR1 models, Figure S2: Results of SiteMap prediction of CCR2 binding sites, Figure S3: Multiple sequence alignment of CC chemokine receptors, Figure S4: CCR2 residues involved in interactions with best-scored compounds obtained in VS using the 6GPX structure, Figure S5: CCR3 residues involved in interactions with best-scored compounds obtained in VS using the inactive-state Robetta model, Table S1: Estimation of allostery-related ChEMBL entries by text-mining (descriptions of bioassays), Table S2: Number of compounds used as final training and testing datasets for NN and GBM models, Table S3: A comparison of the crystal structures and models of chemokine receptors CCR1–6, Table S4: The ROC curves obtained for the different CCR models, Table S5: Results of structure-based virtual screening for CCR2 (6GPX), Table S6: Results of structure-based virtual screening for CCR3 (inactive-state Robetta model), Table S7: Compound activity predictions using the sequential Keras/TensorFlow model of NN, Table S8: Compound activity predictions for CC chemokine receptors using LightGBM, Table S9: Compound activity predictions using LightGBM, Table S10: CCR2 actives—Enamine compounds selected by SBVS assisted by LightGBM, Table S11: CCR2 actives—Enamine compounds selected by SBVS assisted by NN, Table S12: CCR3 actives—Enamine compounds selected by SBVS assisted by LightGBM, Table S13: CCR3 actives—Enamine compounds selected by SBVS assisted by NN.
