# Supplementary material for: Chemokine Receptors—Structure-Based Virtual Screening Assisted by Machine Learning
Source: Pharmaceutics. 2023 Feb 3;15(2):516. doi: 10.3390/pharmaceutics15020516 (PMC9965785; doi:10.3390/pharmaceutics15020516)
Supplement: Supplementary file 1 [file pharmaceutics-15-00516-s001.zip › pharmaceutics-2104855-supplementary.pdf]

Supplementary Materials

Table S1. Estimation of allostery-related ChEMBL entries by text-mining (descriptions of bioassays). 6 main ChEMBL activity categories were shown for the CC receptors datasets (ChEMBL accessed on 3rd Jan 2023). Here, original, uncurated ChEMBL datasets were presented.

**Number of entries in 6 main ChEMBL categories**							
Receptor	**IC50**	**inhibition**	**Ki**	**EC50**	**Activity**	**FC**	**Total**
CCR1	1158	210	197	45	41	20	1671
CCR2	3661	312	1374	19	112	9	5487
CCR3	968	192	417	5	55	n/a	1637
CCR4	1521	149	973	9	44	n/a	2696
CCR5	3869	234	1085	98	173	27	5486
CCR6	365	6	n/a	1	9	n/a	381
**Number of allostery-related entries**							
Receptor	**IC50**	**inhibition**	**Ki**	**EC50**	**Activity**	**FC**	**Total**
CCR1	0	0	0	44	0	20	64
CCR2	1	0	0	0	0	0	1
CCR3	0	0	0	0	0	n/a	0
CCR4	0	0	0	0	0	n/a	0
CCR5	0	0	0	53	3	13	69
CCR6	0	0	n/a	0	0	n/a	0
**% of allostery-related entries in 6 main ChEMBL categories**							
Receptor	**IC50**	**inhibition**	**Ki**	**EC50**	**Activity**	**FC**	**Total**
CCR1	0.0	0.0	0.0	97.8	0.0	100.0	3.8
CCR2	0.0	0.0	0.0	0.0	0.0	0.0	0.0
CCR3	0.0	0.0	0.0	0.0	0.0	n/a	0.0
CCR4	0.0	0.0	0.0	0.0	0.0	n/a	0.0
CCR5	0.0	0.0	0.0	54.1	1.7	48.1	1.3
CCR6	0.0	0.0	n/a	0.0	0.0	n/a	0.0

Table S2. Number of compounds used as final training and testing datasets for NN and GBM models.

Receptor	# of compounds with 'inactives' (training set)	# of compounds without 'inactives' (training set)	# of compounds with 'inactives' (testing set)	# of compounds without 'inactives' (testing set)	Total # of curated compounds (with 'inactives')	Total # of curated compounds (without 'inactives')
CCR1	552	535	237	229	789	764
CCR2	1746	1125	748	482	2494	1607
CCR3	528	518	226	222	754	740
CCR4	926	339	397	146	1323	485
CCR5	2003	1398	858	599	2861	1997
CCR6	240	N/A	103	N/A	343	N/A

Table. S3. A comparison of the crystal structures and models of chemokine receptors CCR1-6.

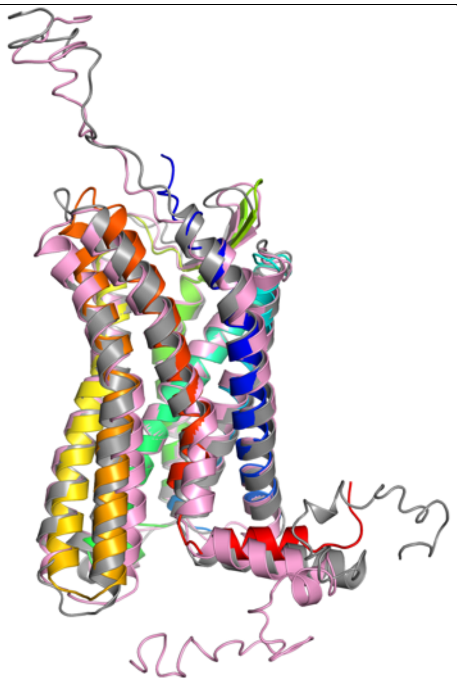

active CCR1 models: blue-to-red - GPCRdb, gray - I-TASSER; and intermediate CCR1 model: pink - GPCRdb

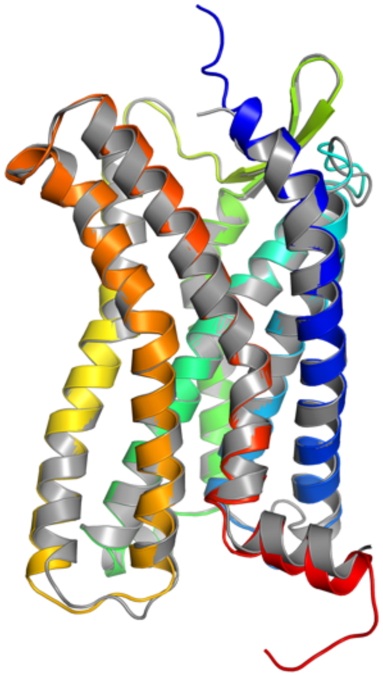

inactive CCR1 models: blue-to-red - GPCRdb, gray - Robetta

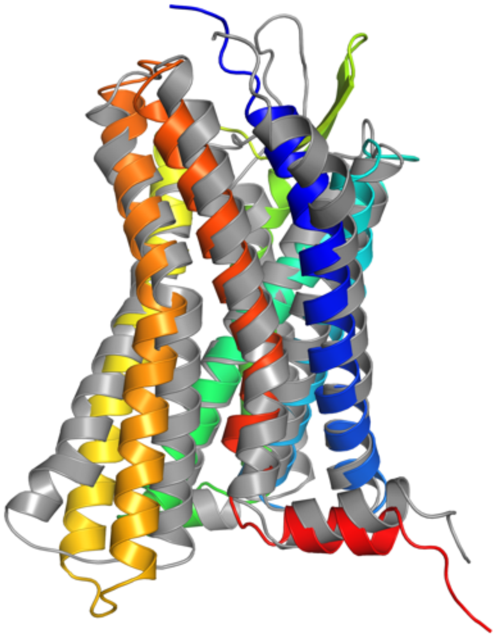

active and intermediate CCR2 models: blue-to-red: inactive GPCRdb, gray - intermediate GPCRdb

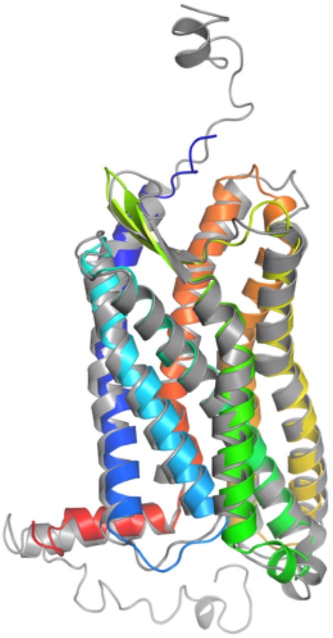

active CCR3 models: blue-to-red - GPCRdb, gray - I-TASSER

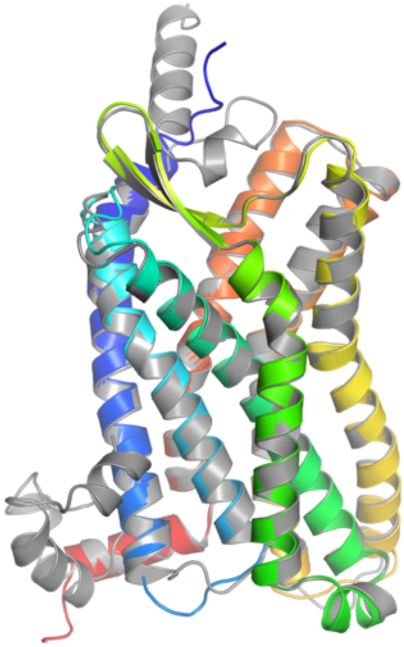

inactive CCR3 models: blue-to-red - GPCRdb, gray - Robetta

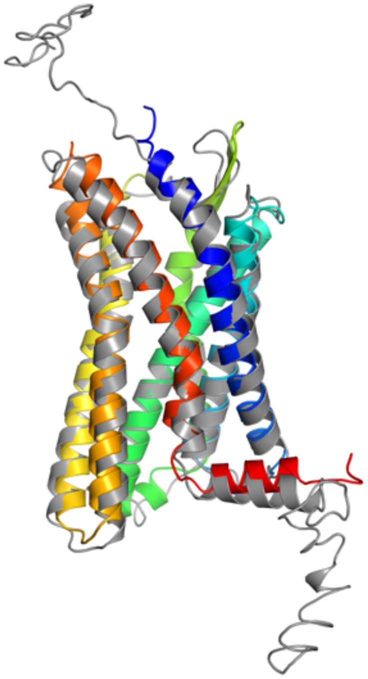

active CCR4 models: blue-to-red - GPCRdb, gray - I-TASSER

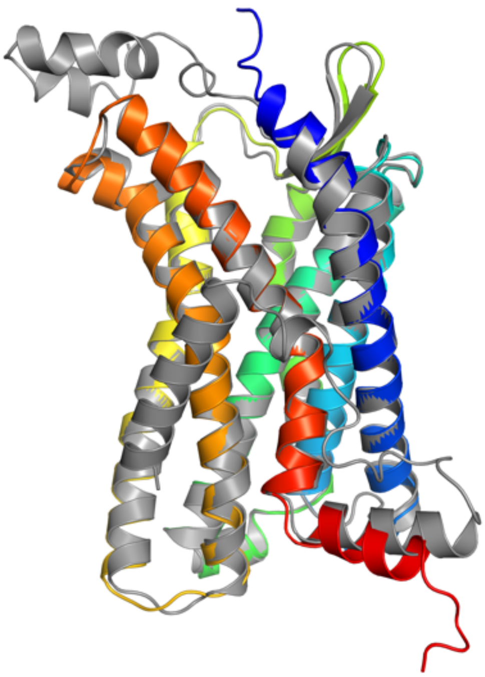

inactive CCR4 models: blue-to-red - GPCRdb, gray - Robetta

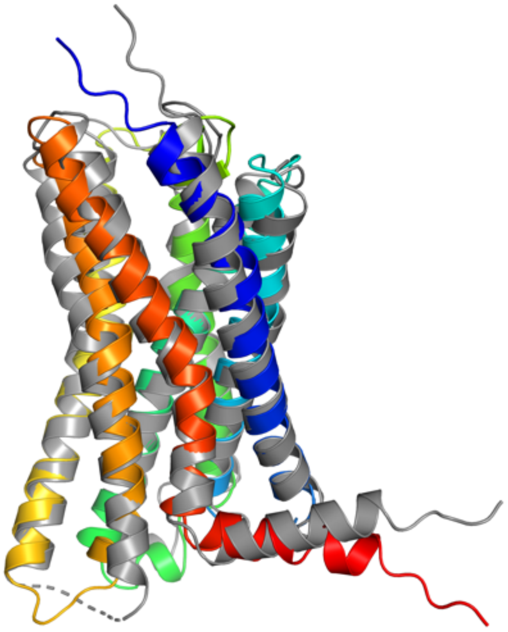

inactive and intermediate CCR6 models: blue-to-red inactive GPCRdb, gray - intermediate GPCRdb

Figure S1. A comparison of the inactive-state CCR1 models: Robetta (gray), GPCRdb (blue-to-red).

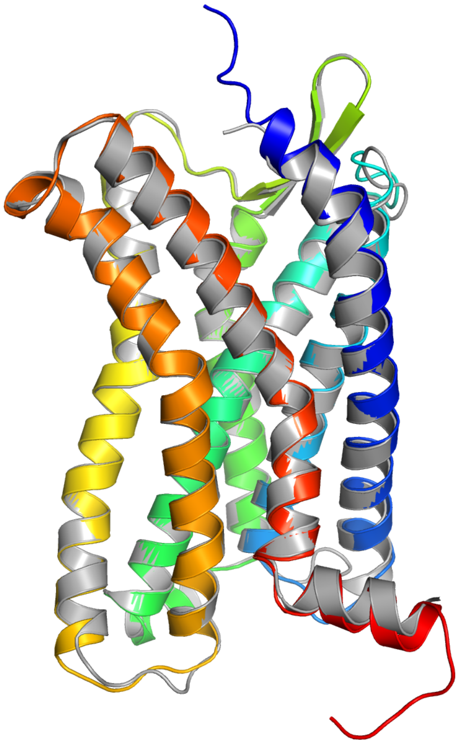

Table S4. The ROC curves obtained for the different CCR models.

<table>
<tr><th colspan="3">CCR1</th></tr>
<tr><th>active</th><th>intermediate</th><th>inactive</th></tr>
<tr>
<td>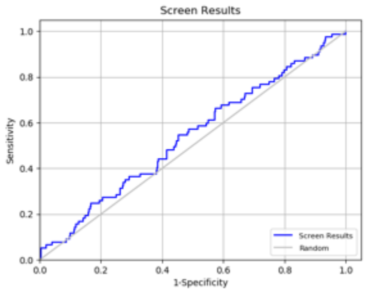

GPCRdb
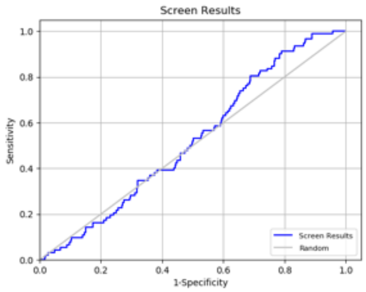

I-TASSER</td>
<td>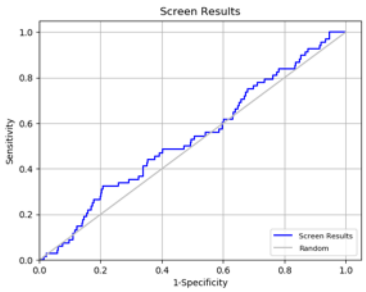

I-TASSER</td>
<td>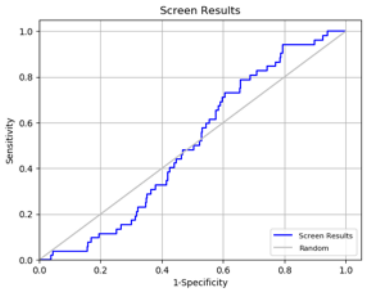

GPCRdb
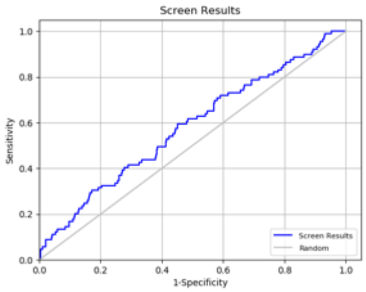

Robetta</td>
</tr>
</table>

<table>
<tr><th colspan="3">CCR2</th></tr>
<tr><th>active</th><th>intermediate</th><th>inactive</th></tr>
<tr><td>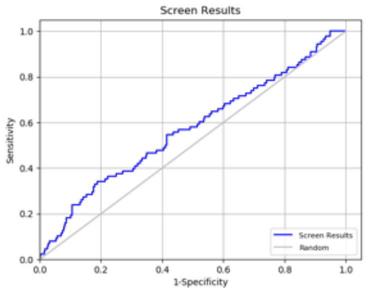

GPCRdb</td><td>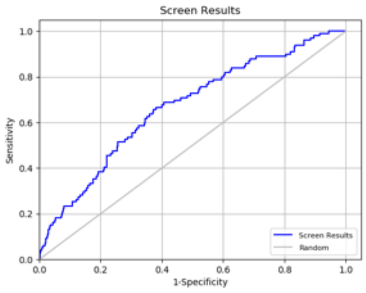

GPCRdb</td><td>—</td></tr>
<tr><th colspan="3">CCR3</th></tr>
<tr><th>active</th><th>intermediate</th><th>inactive</th></tr>
<tr><td>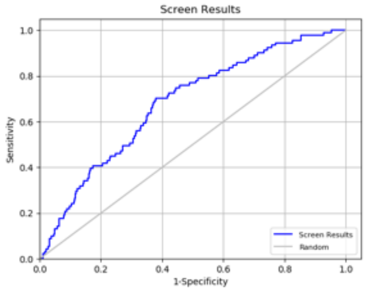

GPCRdb
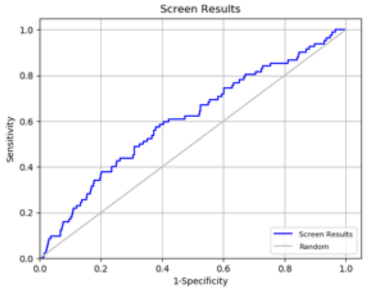

I-TASSER</td><td>—</td><td>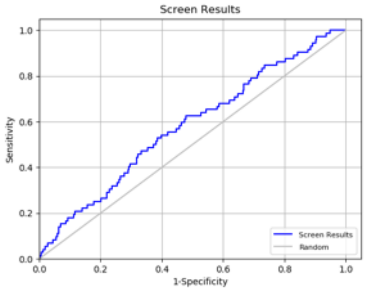

GPCRdb
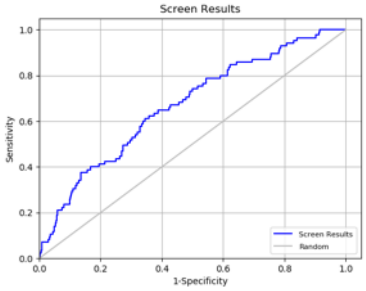

Robetta</td></tr>
<tr><th colspan="3">CCR4</th></tr>
<tr><th>active</th><th>intermediate</th><th>inactive</th></tr>
<tr><td>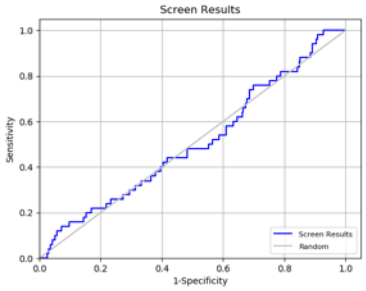

GPCRdb</td><td>—</td><td>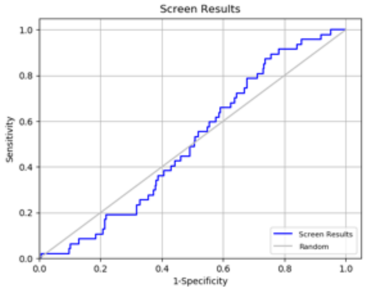

GPCRdb</td></tr>
</table>

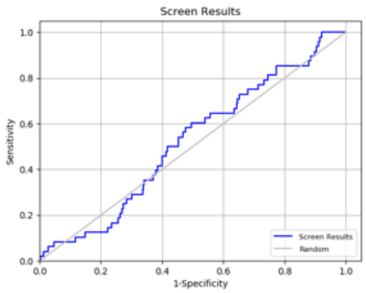 I-TASSER		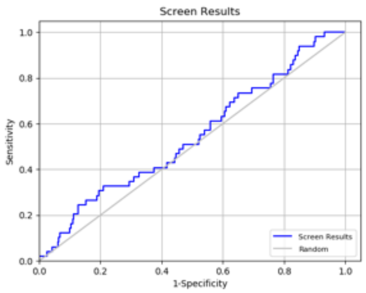 Robetta
CCR6		
active	intermediate	inactive
—	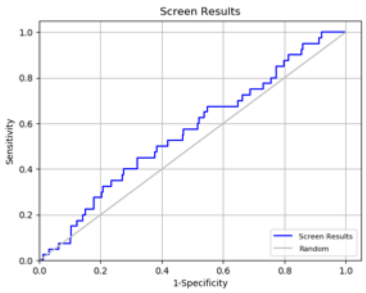 GPCRdb	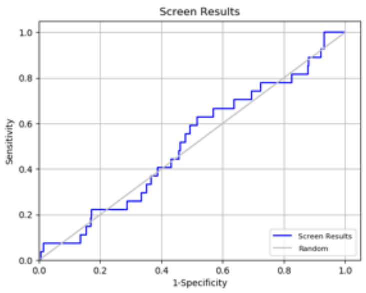 GPCRdb

Figure S2. Results of SiteMap prediction of CCR2 binding sites. The 4MBS ligand and inactive CCR2 Robetta model binding sites; yellow - hydrophobic interactions, blue - hydrogen bond donor, red - hydrogen bond acceptor.

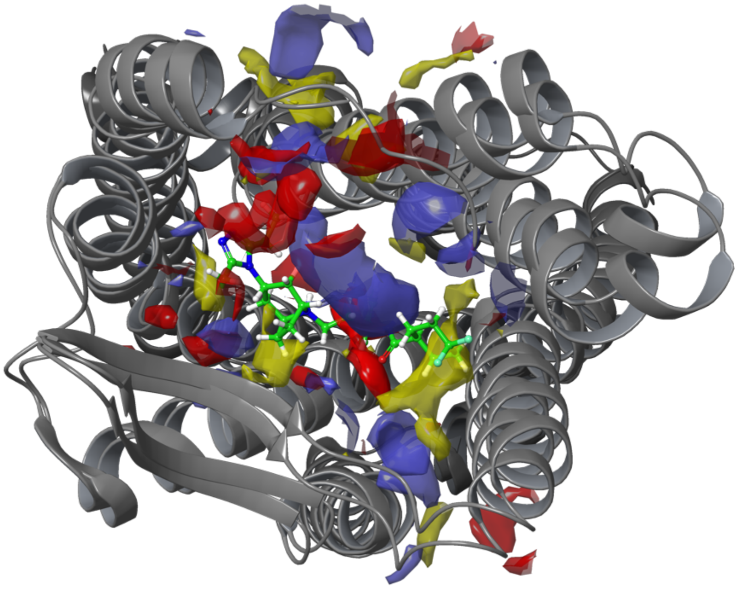

Figure S3. Multiple sequence alignment of CC chemokine receptors. The most conserved residues in each TM helix were depicted and used for residue numbering in the Ballesteros-Weinstein notation.

```
                          1
sp|P32246|CCR1_HUMAN  -MET-------P -----NTTEDYD TTTEFDYGDATP CQKVNERAFGAQ
sp|P41597|CCR2_HUMAN  MLSTSRSRFIRN -----TNESGEE VTTFFDYDYGAP CHKFDVKQIGAQ
sp|P51677|CCR3_HUMAN  -MTTS------L -----DTVETFG TT-SYYDDVGLL CEKADTRALMAQ
sp|P51679|CCR4_HUMAN  -MNPT--DIADT -----TLDESIY SNYYLYESIPKP CTKEGIKAFGEL
sp|P51681|CCR5_HUMAN  ------------ -----MDYQVSS PIYDINYYTSEP CQKINVKQIAAR
sp|P51684|CCR6_HUMAN  -MSGESMNFSDV FDSSEDYFVSVN TSYYSVDSEMLL CSLQEVRQFSRL

                     49            N1.50                      D2.50
sp|P32246|CCR1_HUMAN  LLPPLYSLVFVI GLVGNILVVLVL VQYKRLKNMTSI YLLNLAISDLLF
sp|P41597|CCR2_HUMAN  LLPPLYSLVFIF GFVGNMLVVLIL INCKKLKCLTDI YLLNLAISDLLF
sp|P51677|CCR3_HUMAN  FVPPLYSLVFTV GLLGNVVVVMIL IKYRRLRIMTNI YLLNLAISDLLF
sp|P51679|CCR4_HUMAN  FLPPLYSLVFVF GLLGNSVVVLVL FKYKRLRSMTDV YLLNLAISDLLF
sp|P51681|CCR5_HUMAN  LLPPLYSLVFIF GFVGNMLVILIL INCKRLKSMTDI YLLNLAISDLFF
sp|P51684|CCR6_HUMAN  FVPIAYSLICVF GLLGNILVVITF AFYKKARSMTDV YLLNMAIADILF

                     97                                           R3.50
sp|P32246|CCR1_HUMAN  LFTLPFWIDYKL KDDWVFGDAMCK ILSGFYYTGLYS EIFFIILLTIDR
sp|P41597|CCR2_HUMAN  LITLPLWAHSA- ANEWVFGNAMCK LFTGLYHIGYFG GIFFIILLTIDR
sp|P51677|CCR3_HUMAN  LVTLPFWIHYVR GHNWVFGHGMCK LLSGFYHTGLYS EIFFIILLTIDR
sp|P51679|CCR4_HUMAN  VFSLPFWGYYA- ADQWVFGLGLCK MISWMYLVGFYS GIFFVMLMSIDR
sp|P51681|CCR5_HUMAN  LLTVPFWAHYA- AAQWDFGNTMCQ LLTGLYFIGFFS GIFFIILLTIDR
sp|P51684|CCR6_HUMAN  VLTLPFWAVSHA TGAWVFSNATCK LLKGIYAINFNC GMLLLTCISMDR

                    145                          W4.50
sp|P32246|CCR1_HUMAN  YLAIVHAV--FA LRARTVTFGVIT SIIIWALAILAS MPGLYFSKTQWE
sp|P41597|CCR2_HUMAN  YLAIVHAV--FA LKARTVTFGVVT SVITWLVAVFAS VPGIIFTKCQKE
sp|P51677|CCR3_HUMAN  YLAIVHAV--FA LRARTVTFGVIT SIVTWGLAVLAA LPEFIFYETEEL
sp|P51679|CCR4_HUMAN  YLAIVHAV--FS LRARTLTYGVIT SLATWSVAVFAS LPGFLFSTCYTE
sp|P51681|CCR5_HUMAN  YLAVVHAV--FA LKARTVTFGVVT SVITWVVAVFAS LPGIIFTRSQKE
sp|P51684|CCR6_HUMAN  YIAIVQATKSFR LRSRTLPRSKII CLVVWGLSVIIS SSTFVFNQKYNT

                    193                             P5.50
sp|P32246|CCR1_HUMAN  FTHHTCSLHFPH ES-LREWKLFQA LKLNLFGLVLPL LVMIICYTGIIK
sp|P41597|CCR2_HUMAN  DSVYVCGPYFPR -----GWNNFHT IMRNILGLVLPL LIMVICYSGILK
sp|P51677|CCR3_HUMAN  FEETLCSALYPE DT-VYSWRHFHT LRMTIFCLVLPL LVMAICYTGIIK
sp|P51679|CCR4_HUMAN  RNHTYCKTKYSL NS--TTWKVLSS LEINILGLVIPL GIMLFCYSMIIR
sp|P51681|CCR5_HUMAN  GLHYTCSSHFPY SQ-YQFWKNFQT LKIVILGLVLPL LVMVICYSGILK
sp|P51684|CCR6_HUMAN  QGSDVCEPKYQT VSEPIRWKLLML GLELLFGFFIPL MFMIFCYTFIVK

                    241                         P6.50
sp|P32246|CCR1_HUMAN  ILLRRPNEKK-S KAVRLIFVIMII FFLFWTPYNLTI LISVFQDFLFTH
sp|P41597|CCR2_HUMAN  TLLRCRNEKKRH RAVRVIFTIMIV YFLFWTPYNIVI LLNTFQEFFGLS
sp|P51677|CCR3_HUMAN  TLLRCPSKKK-Y KAIRLIFVIMAV FFIFWTPYNVAI LLSSYQSILFGN
sp|P51679|CCR4_HUMAN  TLQHCKNEKK-N KAVKMIFAVVVL FLGFWTPYNIVL FLETLVELEVLQ
sp|P51681|CCR5_HUMAN  TLLRCRNEKKRH RAVRLIFTIMIV YFLFWAPYNIVL LLNTFQEFFGLN
sp|P51684|CCR6_HUMAN  TLVQAQNSKR-H KAIRVIIAVVLV FLACQIPHNMVL LVTAANLGKMNR

                    289                     P7.50
sp|P32246|CCR1_HUMAN  ECEQSRHLDLAV QVTEVIAYTHCC VNPVIYAFVGER FRKYLRQLFH-R
sp|P41597|CCR2_HUMAN  NCESTSQLDQAT QVTETLGMTHCC INPIIYAFVGEK FRSLFHIALG-C
sp|P51677|CCR3_HUMAN  DCERSKHLDLVM LVTEVIAYSHCC MNPVIYAFVGER FRKYLRHFFH-R
sp|P51679|CCR4_HUMAN  DCTFERYLDYAI QATETLAFVHCC LNPIIYFFLGEK FRKYILQLFKTC
sp|P51681|CCR5_HUMAN  NCSSSNRLDQAM QVTETLGMTHCC INPIIYAFVGEK FRNYLLVFFQ-K
sp|P51684|CCR6_HUMAN  SCQSEKLIGYTK TVTEVLAFLHCC LNPVLYAFIGQK FRNYFLKILKDL

                    337
sp|P32246|CCR1_HUMAN  RVAVHLVKWLPF LSVDRLERVSST SPS----TGEHE LSAGF-------
sp|P41597|CCR2_HUMAN  RIAPLQKPVCGG PGV-RPGKNVKV TTQGLLDGRGKG KSIGRAPEASLQ
sp|P51677|CCR3_HUMAN  HLLMHLGRYIPF LPSEKLERTSSV SPS----TAEPE LSIVF-------
sp|P51679|CCR4_HUMAN  RGLFVLCQYCGL LQIYSADTPSSS YTQSTMDHDLHD AL----------
sp|P51681|CCR5_HUMAN  HIAKRFCKCCSI FQQEAPERASSV YTRST---GEQE ISVGL-------
sp|P51684|CCR6_HUMAN  WCVRRKYKSSGF SCAGRYSENISR QTSET---ADND NASSFTM-----

                    385
sp|P32246|CCR1_HUMAN  -----
sp|P41597|CCR2_HUMAN  DKEGA
sp|P51677|CCR3_HUMAN  -----
sp|P51679|CCR4_HUMAN  -----
sp|P51681|CCR5_HUMAN  -----
sp|P51684|CCR6_HUMAN  -----
```

Figure S4. CCR2 residues involved in interactions with best-scored compounds obtained in VS using the 6KPX structure. The receptor was shown in the blue-to-red color scheme. Compounds were shown in grey with polar contacts indicated with yellow dashed lines. Residues were numbered according to the Ballesteros-Weinstein numbering scheme (**Lys38 -> Lys1.28**; Tyr49->Tyr1.39; **Asn175->Asn4.60; Thr179->Thr4.64**; His202->His5.38; Arg206->Arg5.42; Tyr259->Tyr6.51; **Gln288->Gln7.36**; **Glu291->Glu7.39**; Thr292-> Thr7.40). Residues involved in polar interactions with ligand in the 6KPX structure were marked in red. The 6KPX ligand was shown in sticks and in green.

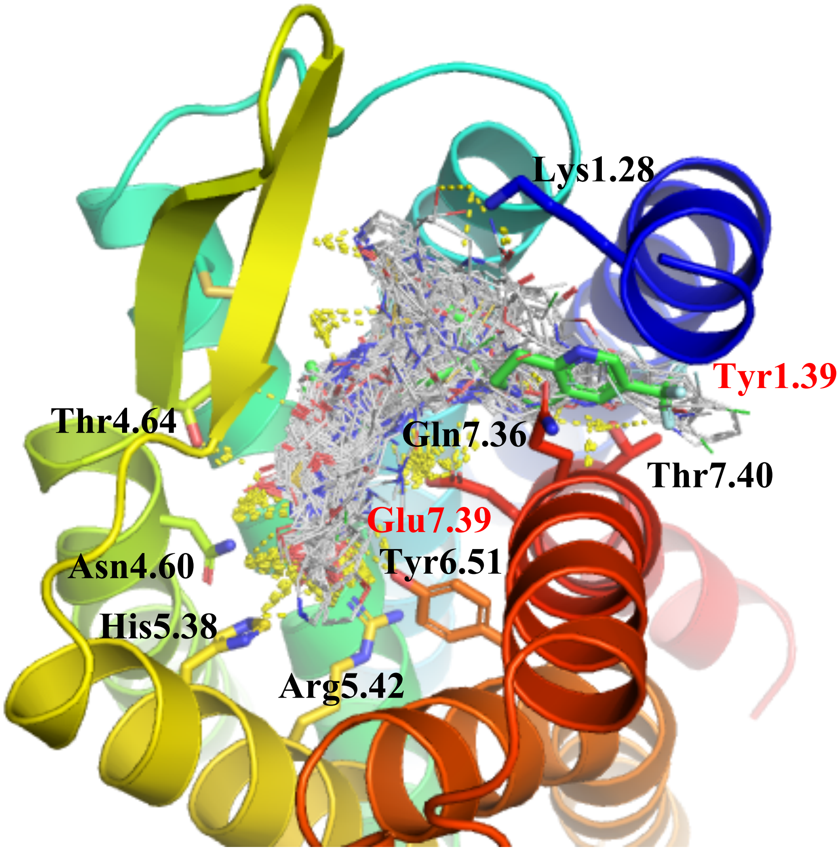

Figure S5. CCR3 residues involved in interactions with best-scored compounds obtained in VS using the inactive-state Robetta model. The receptor was shown in the blue-to-red color scheme with dark-blue N-terminus capping the receptor from the extracellular side. Compounds were shown in grey with polar contacts indicated with yellow dashed lines. Residues were numbered according to the Ballesteros-Weinstein numbering scheme (Thr29->Thr1.27; **Arg30->Arg1.28**; Tyr93->Tyr2.63; Ser110->Ser3.29; Tyr113->Tyr3.32; His114->His3.33; **Glu168->Glu4.60**; **Tyr255->Tyr6.51**; Asp280->Asp7.32; Glu287->Glu7.39; Tyr291->Tyr7.43). Residues that were also involved in ligand interactions in CCR2 (see Fig. S4) were marked with red. The 6KPX ligand was shown in sticks and in green.

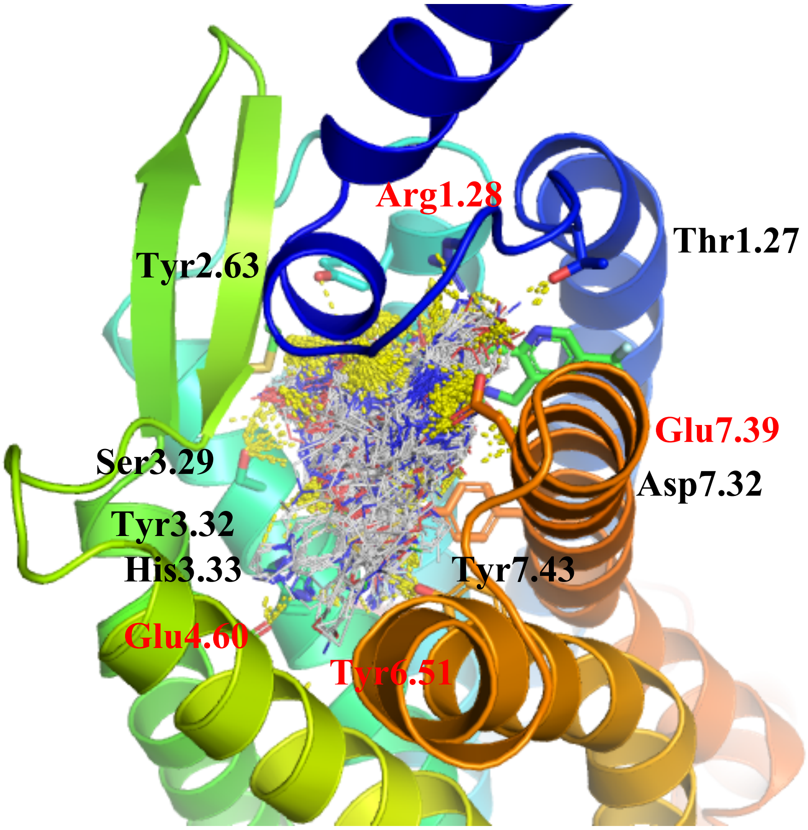

Table S5. Results of structure-based virtual screening for CCR2 (6GPX). Residues numbering in accordance with Supplementary Fig. S4.

Cluster ID	Number of cluster members	SMILES of medoid	2D structure of medoid and its interactions	IUPAC name	synonyms	XP GScore	Known protein targets incl. analogs
1	1	OC[C@@H]1C[C@@H](C1)Nc(n2)c(nc(c23)ncc3)N[C@@H]4C[C@@H](C4)CO	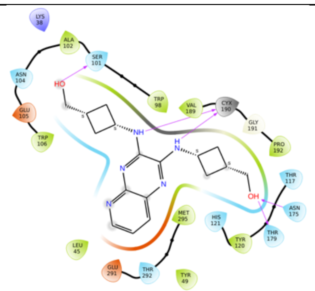	[3-[[3-[[3-(hydroxymethyl)cyclobutyl]amino]pyrido[2,3-b]pyrazin-2-yl]amino]cyclobutyl]methanol	—	-8.614	Transporters (ZINC3206141)

2	3	O[C@@H]1CC[C@H](CC1)[NH2+]Cc2cn(-c3ccccc3)nc2-c4cccnc4	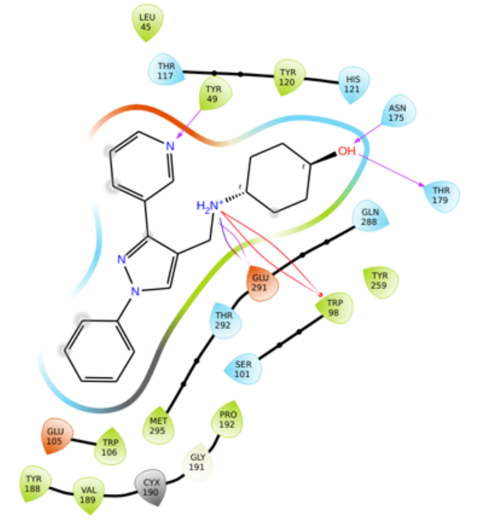	4-[(1-phenyl-3-pyridin-3-ylpyrazol-4-yl)methylamino]cyclohexan-1-ol	ZINC61003515<br>AKOS034182580<br>Z323167464<br>4-({[1-phenyl-3-(pyridin-3-yl)-1H-pyrazol-4-yl]methyl}amino)cyclohexan-1-ol<br>1355560-09-6	-8.220	Influenza A NS1 protein (CHEMBL1450506)
3	1	c1ncccc1C[NH2+]Cc2cn[nH]c2-c(c3)oc(c34)cccc4	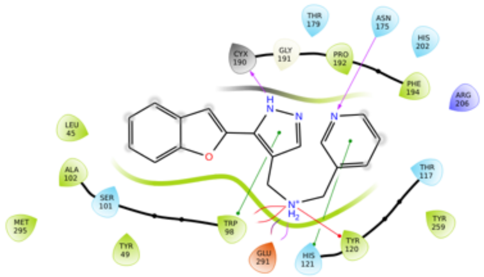	*N*-[[5-(1-benzofuran-2-yl)-1*H*-pyrazol-4-yl]methyl]-1-pyridin-3-ylmethanamine	ZINC97159732<br>AKOS034612768<br>Z823355126<br>{[3-(1-benzofuran-2-yl)-1H-pyrazol-4-yl]methyl}[(pyridin-3-yl)methyl]amine<br>1798742-45-6	-8.673	Glycylpeptide N-tetradecanoyltransferase (ZINC97159732)
4	18	Cc1ccc(cc1C)C(=O)NCC(=O)N2CCC(CC2)CCc3ccc(O)cc3	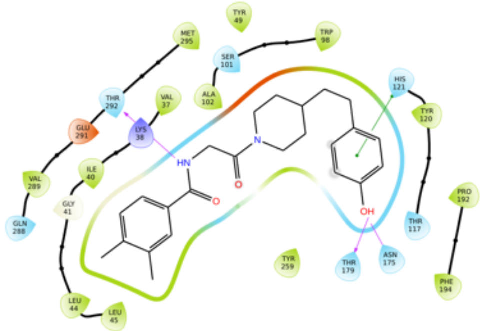	*N*-[2-[4-[2-(4-hydroxyphenyl)ethyl]piperidin-1-yl]-2-oxoethyl]-3,4-dimethylbenzamide	ZINC1299071<br>AKOS008088632	-8.525	Lipid Storage Modulators in Drosophila S3 Cells (CHEMBL1606135)
5	13	Fc1ccc(cc1)C(=O)NCCNC(=O)COc(cc2)cc(c23)cccc3	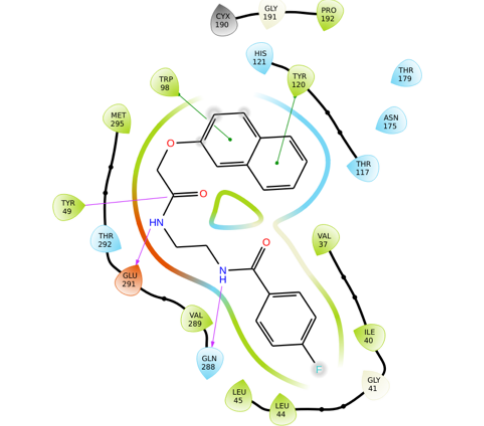	4-fluoro-*N*-[2-[(2-naphthalen-2-yloxyacetyl)amino]ethyl]benzamide	4-fluoro-N-(2-{[(naphthalen-2-yloxy)acetyl]amino}ethyl)benzamide<br>ZINC2850976<br>STK232650<br>AKOS005420715	-8.162	Menin-MLL Interactions in Leukemias (CHEMBL1462783)

<table>
<tr><td>6</td><td>4</td><td>Fc1ccc(cc1)C(=O)N2CCC(CC2)C(=O)NCCc(c3)[nH+]c(n34)cccc4</td><td>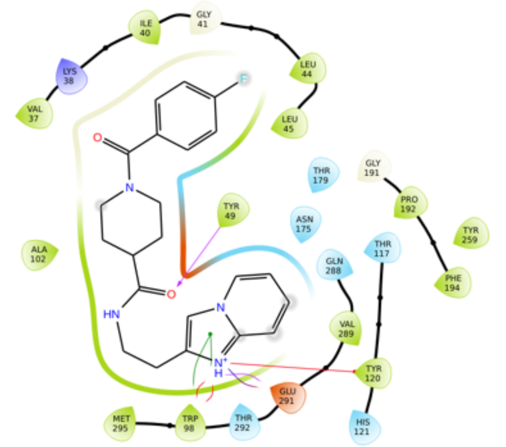
</td><td>1-(4-fluorobenzoyl)-N-(2-imidazo[1,2-a]pyridin-2-ylethyl)piperidine-4-carboxamide</td><td>ZINC11534785
AKOS033878005
Z237159726
1-(4-fluorobenzoyl)-N-(2-{imidazo[1,2-a]pyridin-2-yl}ethyl)piperidine-4-carboxamide
949308-57-0</td><td>-8.315</td><td>L3MBTL3 (SEA predictions for ZINC11534785)</td></tr>
<tr><td>7</td><td>6</td><td>O=C1NC(=O)N[C@]1(C)c2cccc(c2)NC(=O)c3cccc(c3)OCC(=O)[O-]</td><td>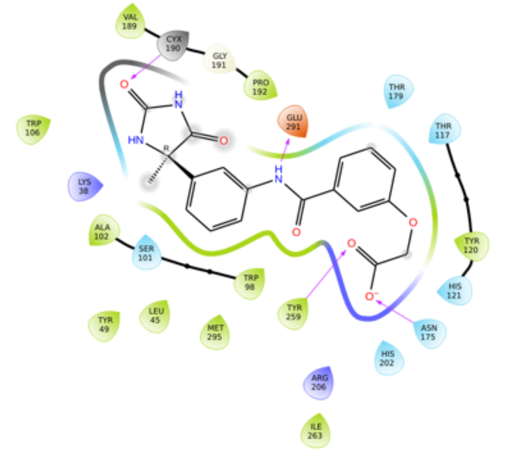
</td><td>—</td><td>ZINC539574927</td><td>-8.987</td><td>FABP4 (SEA predictions for ZINC539574927)</td></tr>
<tr><td>8</td><td>10</td><td>C[C@@H]1CC[C@@H](C(=O)N1)NC(=O)c2ccc(cc2)NC(=O)Nc3ccccc3</td><td>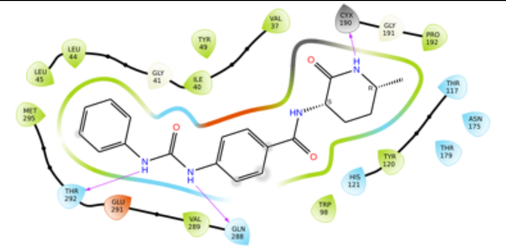</td><td>—</td><td>—</td><td>-8.190</td><td>—</td></tr>
<tr><td>9</td><td>52</td><td>Fc1ccc(cc1F)NC(=O)CC[C@@H](C2)C(=O)Nc(c23)cccc3</td><td>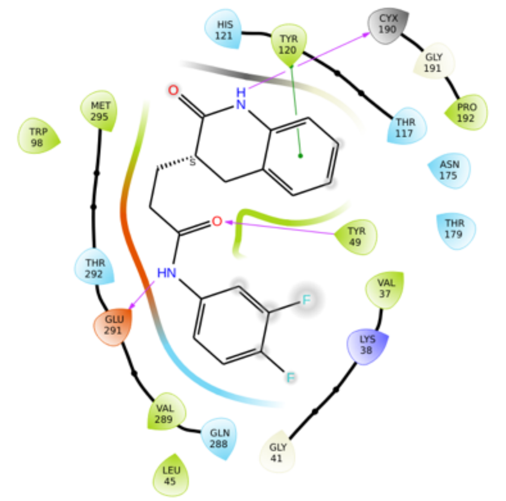
</td><td>N-(3,4-difluorophenyl)-3-[(3R)-2-oxo-3,4-dihydro-1H-quinolin-3-yl]propanamide</td><td>ZINC14898761</td><td>-8.099</td><td>Peptide deformylase (SEA predictions for ZINC14898761)</td></tr>
</table>

10	2	c1ccccc1[C@H]([NH+](C)C)CNC(=O)CSc2nnc(o2)-c3ccc(Cl)cc3	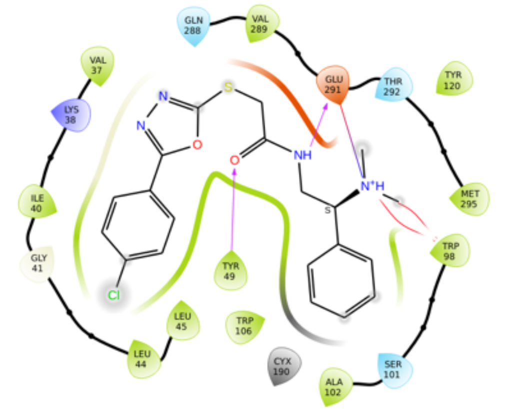	2-[[5-(4-chlorophenyl)-1,3,4-oxadiazol-2-yl]sulfanyl]-*N*-[(2*R*)-2-(dimethylamino)-2-phenylethyl]acetamide	ZINC3318021	-8.293	—
11	204	c1cccc(c12)c(c[nH]2)C[C@H](C([O-])=O)NC(=O)CCCc(c[nH]3)c(c34)cccc4	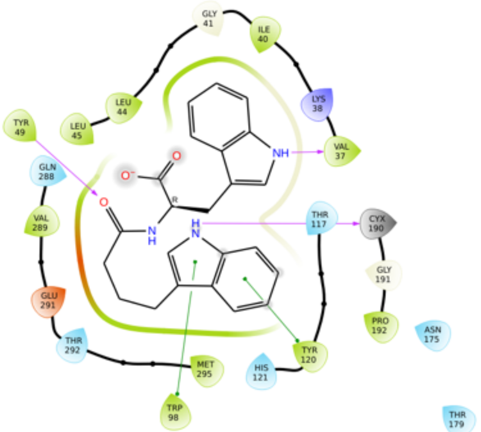	(2*S*)-3-(1*H*-indol-3-yl)-2-[4-(1*H*-indol-3-yl)butanoylamino]propanoic acid	406726-77-0<br>(S)-3-(1H-Indol-3-yl)-2-(4-1H-indol-3-yl-butyrylamino)-propionic acid<br>Indolebutyric acid-tryptophan conjugate<br>IBA-L-Trp<br>MLS001216373<br>SCHEMBL1233360<br>CHEMBL1698660<br>DTXSID10349336<br>HMS2850B20<br>ZINC4090324<br>STL529945<br>AKOS032434392<br>SMR000544296<br>N-[4-(1H-indol-3-yl)butanoyl]-L-tryptophan<br>L-tryptophan, N-[4-(1H-indol-3-yl)-1-oxobutyl]-L-<br>Tryptophan, N-[4-(1H-indol-3-yl)-1-oxobutyl]-(9CI)<br>(S)-3-(1H-Indol-3-yl)-2-[4-(1H-indol-3-yl)butyrylamino]propionic acid	-9.524	malarial parasite plastid (CHEMBL1698660)
12	4	[O-]C(=O)c(c1)oc(c12)ccc(c2)NC(=O)c3ccnn3C4CCCC4	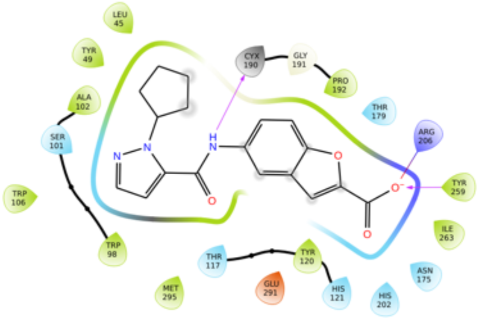	5-[(2-cyclopentylpyrazole-3-carbonyl)amino]-1-benzofuran-2-carboxylic acid	AKOS026493400<br>ZINC262730961<br>Z1991757892<br>5-(1-cyclopentyl-1H-pyrazole-5-amido)-1-benzofuran-2-carboxylic acid	-8.204	—

13	4	c1ncccc1NC(=O)C(=O)N2CCC(CC2)c(c[nH]3)c(c34)cccn4	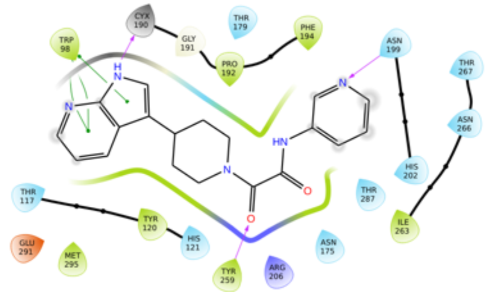	2-oxo-*N*-pyridin-3-yl-2-[4-(1*H*-pyrrolo[2,3-b]pyridin-3-yl)piperidin-1-yl]acetamide	AKOS030723040<br><br>ZINC189084207	-8.056	—
14	5	c1cccc(c12)c(=O)[nH]c(n2)C[N@@H+]3CC[C@@H](CC3)c(c[nH]4)c(c45)cccc5	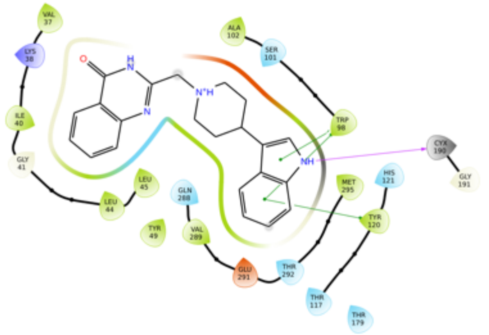	2-[[4-(1*H*-indol-3-yl)piperidin-1-yl]methyl]-3*H*-quinazolin-4-one	ZINC57383144<br><br>AKOS033829044<br><br>Z225098374<br><br>2-{[4-(1H-indol-3-yl)piperidin-1-yl]methyl}-3,4-dihydroquinazolin-4-one<br><br>930849-57-3	-8.515	—
15	1	COc1ccc(cc1)C[N@@H+]2CCC[C@@H](C2)n3ncc(cc3=O)N4CCC(O)CC4	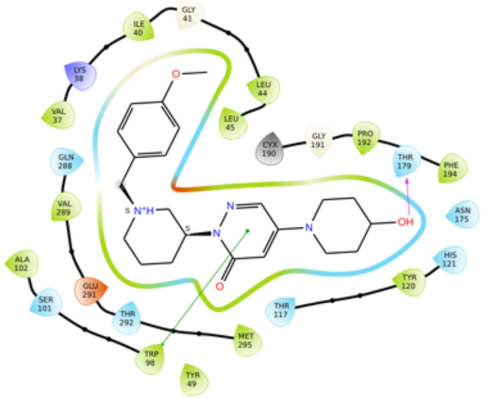	5-(4-hydroxypiperidin-1-yl)-2-[(3*R*)-1-[(4-methoxyphenyl)methyl]piperidin-3-yl]pyridazin-3-one	ZINC328603313	-8.435	—
16	1	FC(F)(F)Oc1ccc(cc1)-c2cc(n[nH]2)C(=O)N3CCC[C@H]3Cn4cncn4	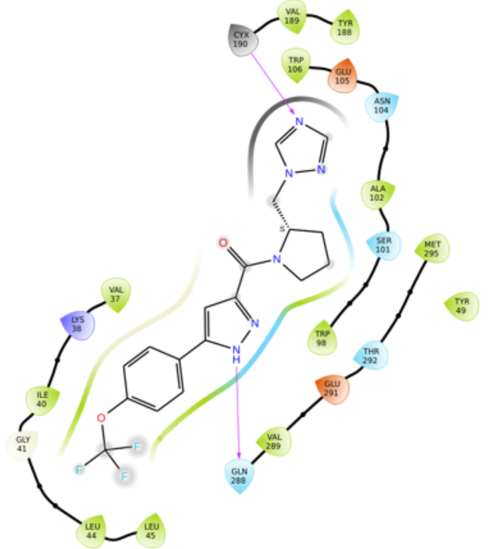	—	—	-8.664	—

17	3	CCn1cc(cn1)CC(=O)N2CC[C@@H](C2)c3cc(=O)[nH]c(n3)N(C)C	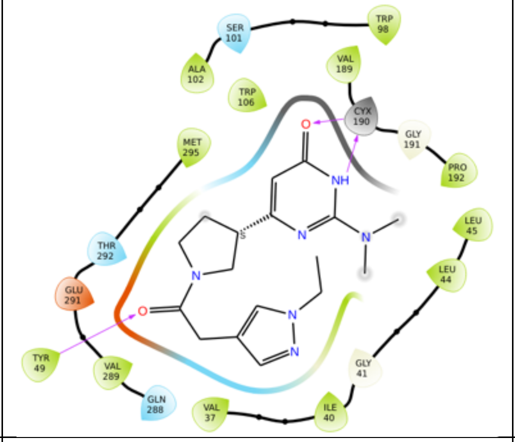	—	ZINC864837158	-8.209	—	
18	4	O=C(Cc2ccc(N1CCCC1=O)cc2)NC4CC(Cn3ccnc3)CC4O	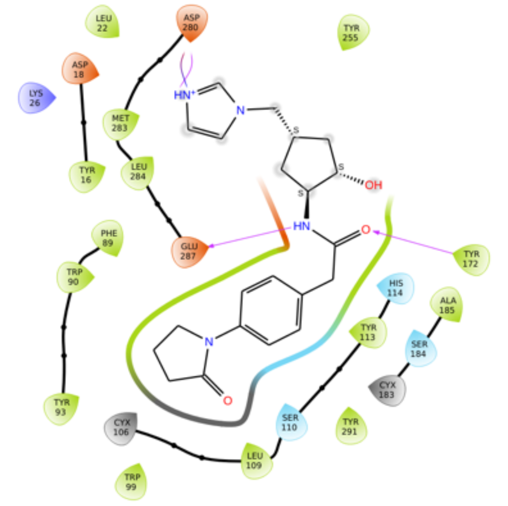	-	-	-8.309	—	
19	116	Cc1sc(C(=O)[O-])cc1C[N@H+](CC)[C@@H]2CCN(C2=O)c3ccccc3	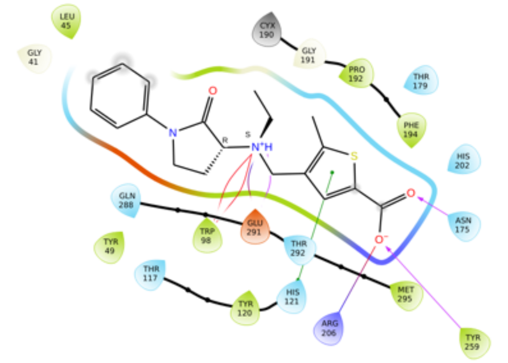	—	ZINC565721925	-9.238	—	
20	3	NC(=O)C[C@@]1(O)CCCN(C1)C(=O)CCc2ccc(F)cc2F	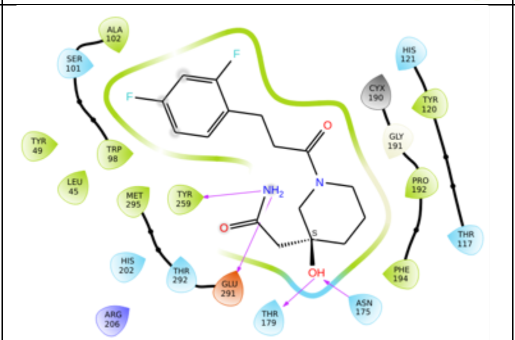	—	ZINC644004667	-8.385	—	

21	1	COC(=O)[C@H]1C[C@@H](O)CN(C1)C(=O)CCC(=O)Nc(cc2)nc(c2=3)=[NH+]C(N3)=O	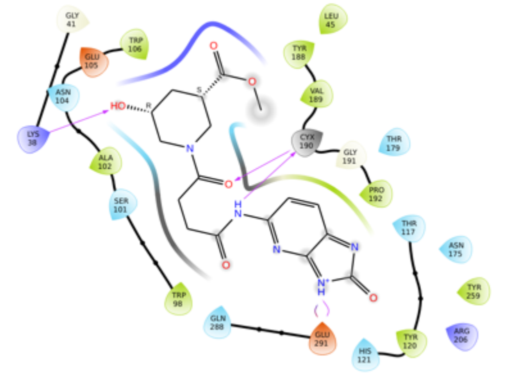	—	—	-8.339	—
22	4	NC(=O)c1cccc(c1)COC(=O)c(c2)c(=O)[nH]c(c23)CCCC3	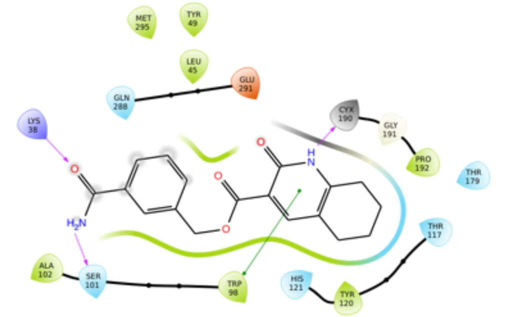	(3-carbamoylphenyl)methyl 2-oxo-5,6,7,8-tetrahydro-1*H*-quinoline-3-carboxylate	AKOS033127504<br><br>Z1168997708<br><br>(3-carbamoylphenyl)methyl 2-oxo-1,2,5,6,7,8-hexahydroquinoline-3-carboxylate	-8.816	—

Table S6. Results of structure-based virtual screening for CCR3 (inactive-state Robetta model). Residues numbering in accordance with Fig. S6.

Cluster ID	Number of cluster members	SMILES of medoid	2D structure of medoid and its interactions	IUPAC name	synonyms	XP GScore
1	1	C1CCCc(n12)[nH+]c(c2)CNc(c(c34)CCC4)nc(n3)-c5ccccn5	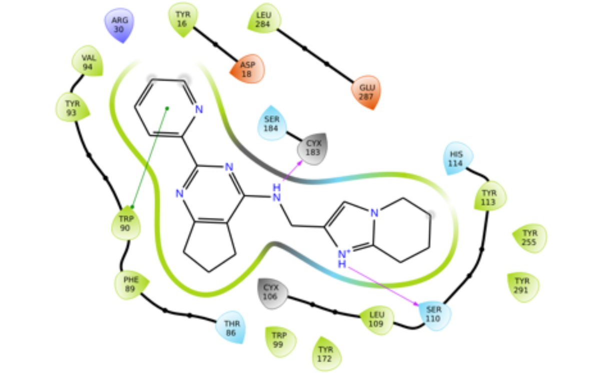	—	—	-10.986
2	1	C1CCCc(c12)c(ccc2)NC(=O)Cc(c3)[nH+]c(n34)cccc4	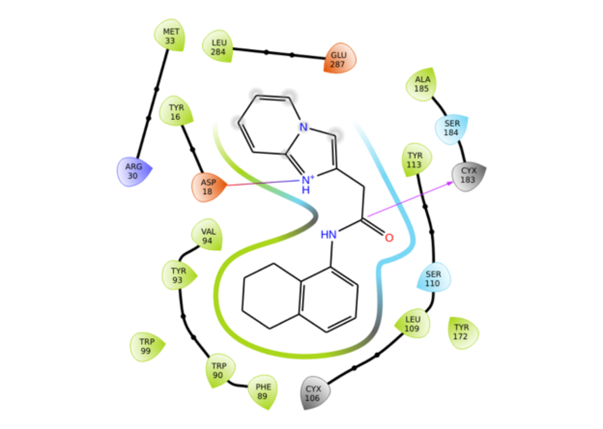	—	ZINC138280841	-10.631

3	1	[NH3+]CCOc1ccc(cc1)NC(=O)C(=CC=2)CC(C23)=NC(=O)N3C4CCCC4	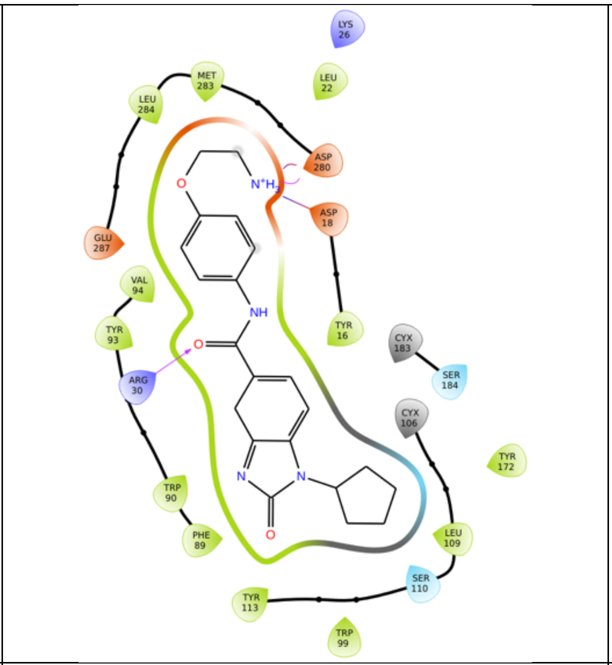	—	—	-10.342
4	1	C1C[N@@H+](C)C[C@@H]([C@H]12)CCC[N@H+]2CCC(=O)Nc(s3)c(C#N)c(c34)CCCCC4	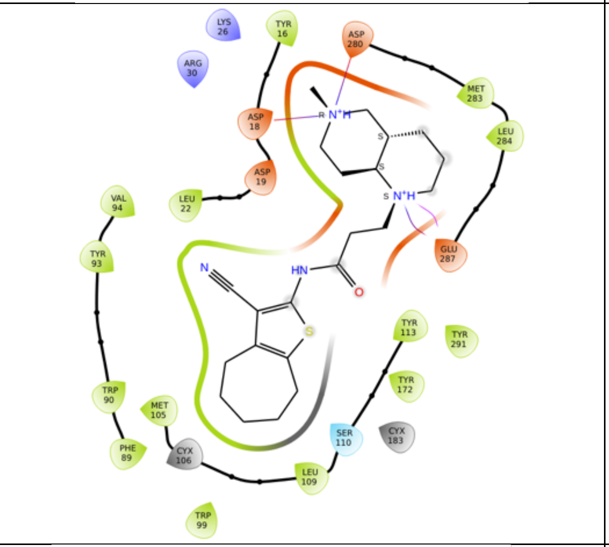	3-[(4aR,8aR)-6-methyl-2,3,4,4a,5,7,8,8a-octahydro-1,6-naphthyridin-1-yl]-N-(3-cyano-5,6,7,8-tetrahydro-4H-cyclohepta[b]thiophen-2-yl)propanamide	ZINC89375274, other isomers: ZINC89375276, ZINC107528310, ZINC229962152	-11.014
5	1	[C@@H]12[C@H]3[C@@H]4[C@@H]5[C@H](C3)[C@H]2[C@@H](C5)[C@@H]4[C@]1(O)C[N@H+]6CCC[C@H](C6)N7CCCC7=O	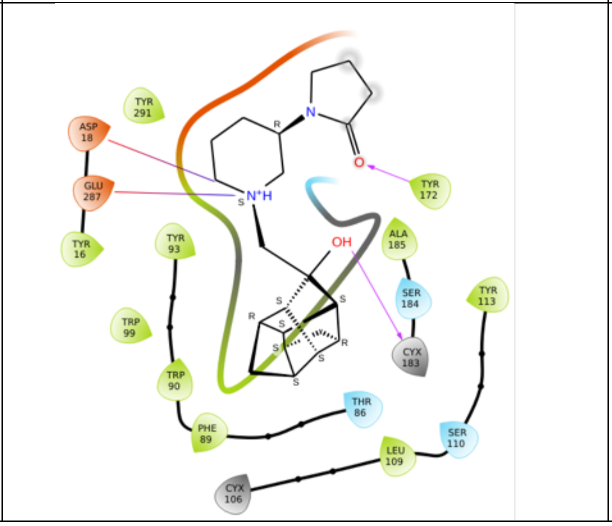	—	—	-10.565

6	1	C[C@@H]1C[C@H]1c2ccc(o2)[C@@H]3C[C@@H]3C(=O)n4ccc(cc4)=[NH+]CC(F)(F)F	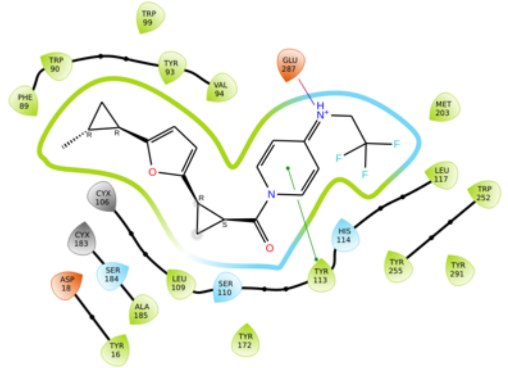	—	—	-11.091
7	18	[NH3+][C@@H]1CCC[C@@H](C1)C(=O)N2CCC(CC2)c(c[nH]3)c(c34)cccn4	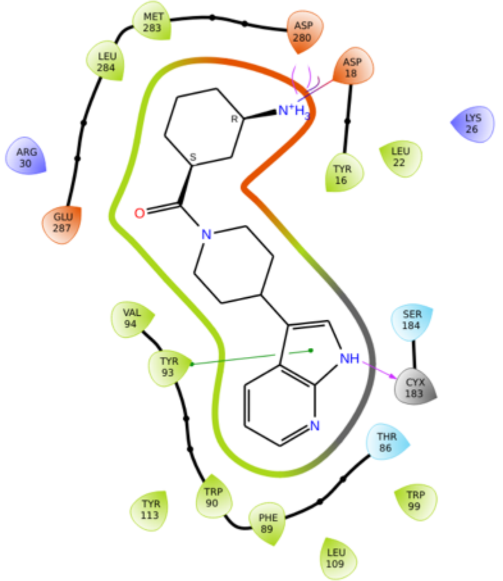	—	—	-11.181
8	4	[NH3+][C@@H](C1)[C@H]([C@H]12)C[C@@H](C2)C(=O)NCc(c3)c(=O)[nH]c(c34)CCCC4	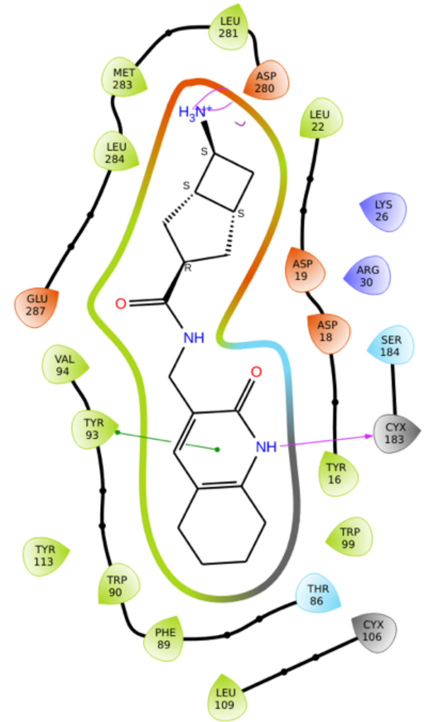	—	—	-11.554

9	36	C1[NH2+]CC[C@@H]1CCC(=O)Nc(n2)sc(c23)C[C@@H](CC3)c4ccccc4	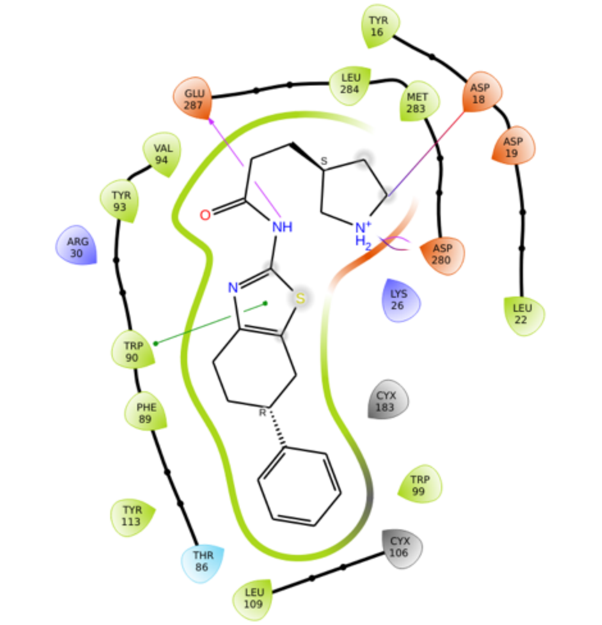	—	—	-10.367
10	264	CNC(=O)C[C@@]1(O)CCC[N@H+](C1)Cc2ccccc2C(F)(F)F	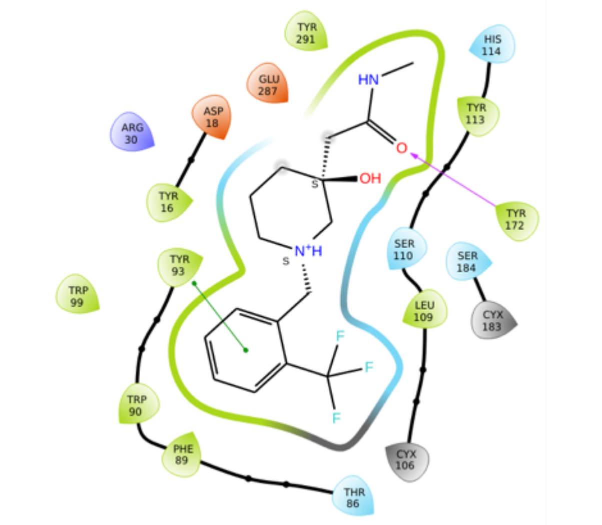	—	—	-10.931
11	35	Cn1cc(cn1)C[N@@H+]2C[C@H](O)C[C@H]2c3cccc(c3)C(F)(F)F	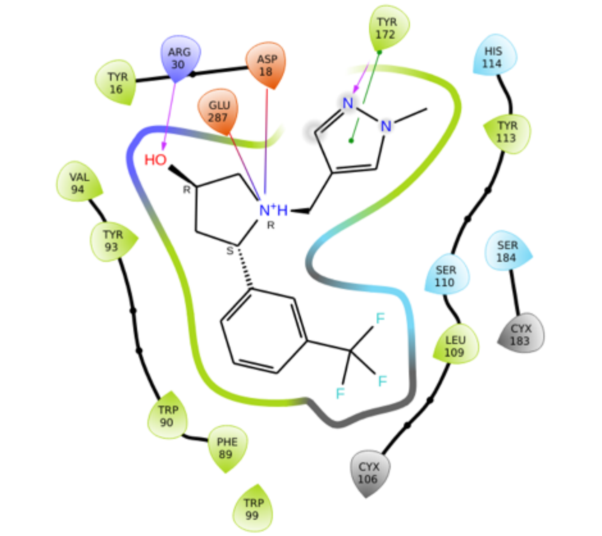	—	—	-10.871

12	2	FC(F)(F)c1cc([nH]n1)[C@@H]2CCC[N@@H+](C2)CCCc3cn[nH]c3	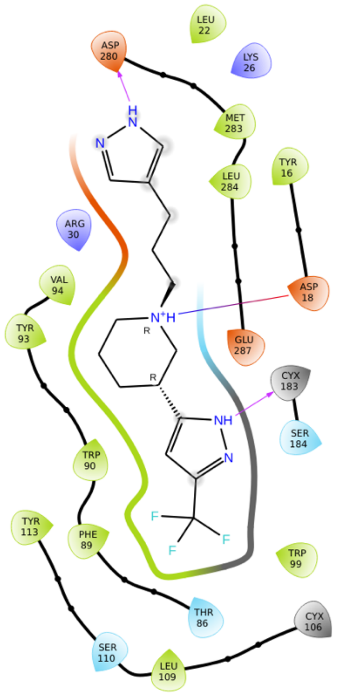	—	—	-11.392
13	11	C[NH+](C)Cc1cc(ccn1)O[C@@H]2CC[N@@H+](C2)Cc(ccc3)n(c34)ccn4	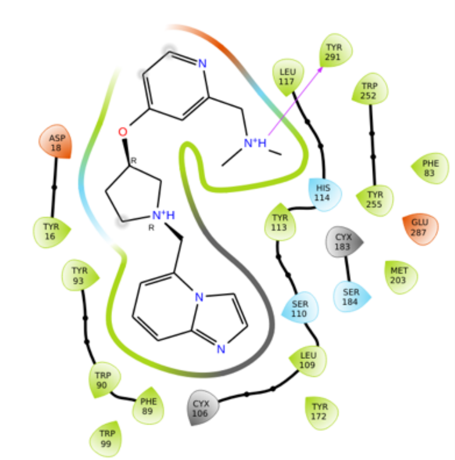	—	—	-11.304
14	39	C1CC1NC(=O)[C@@H](C)[N@@H+]2CC[C@@H](CC2)c(c3)[nH]c(c34)cccc4	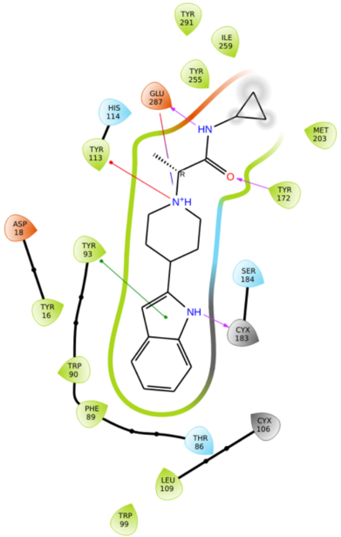	—	—	-11.479

15	2	COc1ccc(cc1Cl)CNc2cc(C(=O)N)ccc2C[NH+](C)C	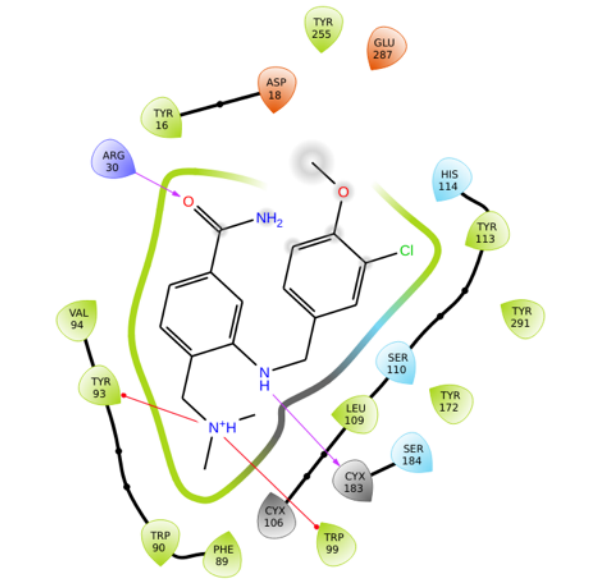	—	—	-11.280
16	4	CC(C)[C@H]1OCCC[C@@H]1Nc2cc(ncn2)NCCn3cc[nH+]c3	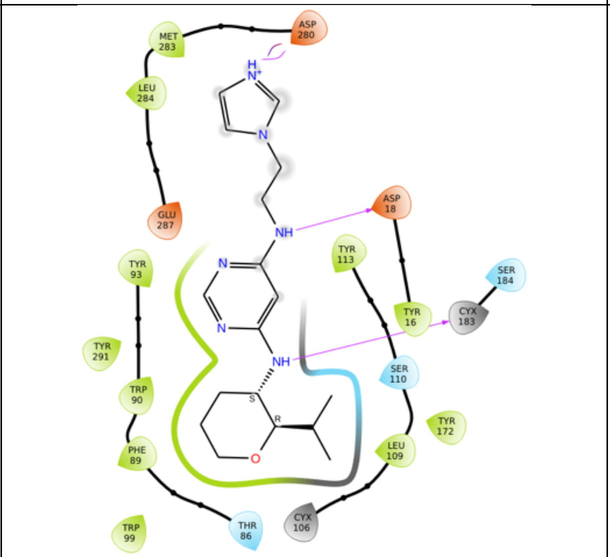	—	—	-11.216
17	3	CC(C)N1CCC[C@H](C1=O)[N@@H+](C)CC2CCN(CC2)c3cc[nH+]cc3	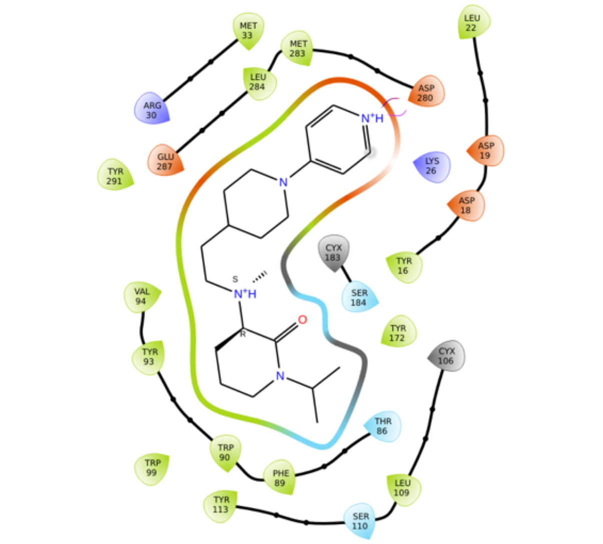	—	—	-10.706

<table>
<tr><th></th><th></th><th></th><th></th><th></th><th></th><th></th></tr>
<tr><td>18</td><td>21</td><td>c1[nH+]ccn1C[C@H](C[C@@H]2O)C[C@@H]2NC(=O)Cc3ccc(cc3)N4CCCC4=O</td><td>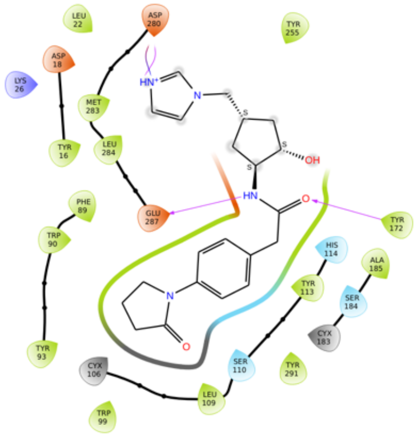
</td><td>—</td><td>—</td><td>-10.823</td></tr>
<tr><td>19</td><td>5</td><td>CC(C)(C)c1ccc(cc1)-c2noc(n2)CC[C@H]3CC[NH2+]C3</td><td>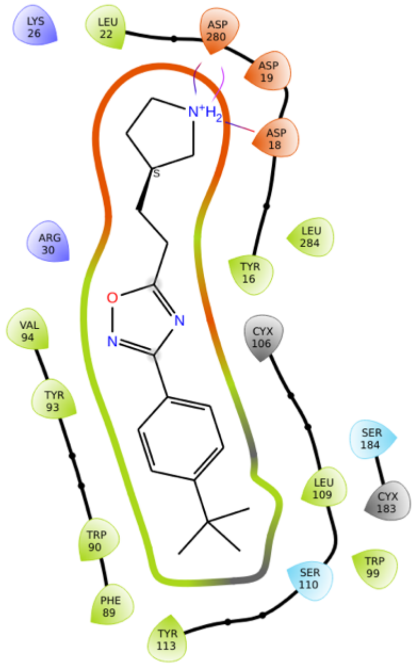
</td><td>—</td><td>—</td><td>-10.959</td></tr>
<tr><td>20</td><td>2</td><td>Fc1cccc(F)c1CNC(=CN=2)CC(C23)=CN=[NH+]3</td><td>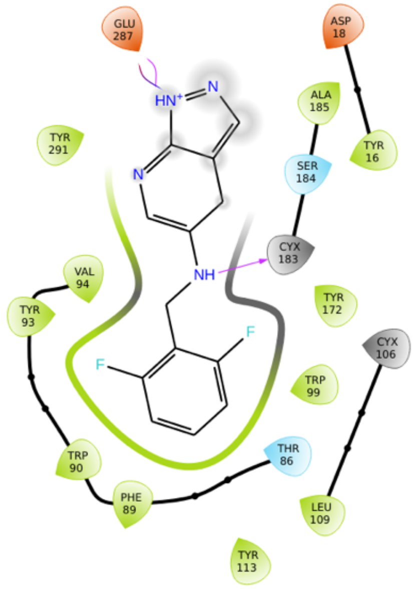
</td><td>—</td><td>—</td><td>-10.667</td></tr>
</table>

21	4	Fc1ccccc1[C@@H](C)C(=O)Nc(cc2)c(F)c(c23)CC[NH2+]C3	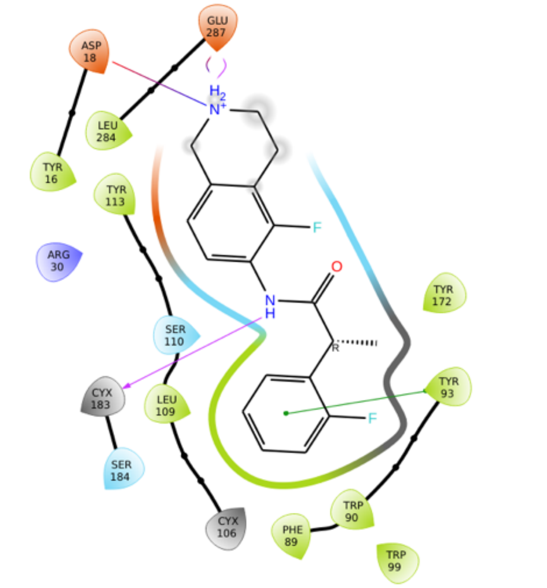	—	—	-10.533
22	4	n1cncn1CCNc(ncn2)c(c23)c(cs3)-c4ccccc4	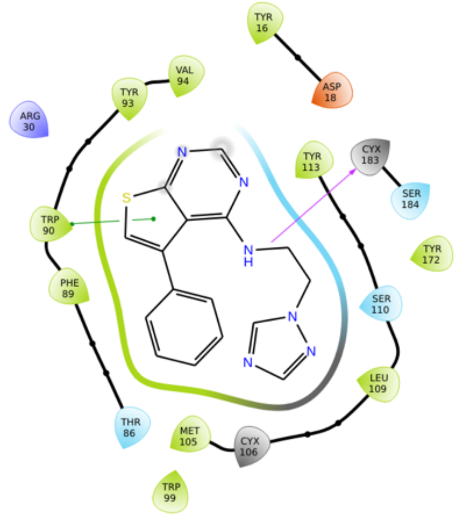	5-phenyl-N-[2-(1H-1,2,4-triazol-1-yl)ethyl]thieno[2,3-d]pyrimidin-4-amine	ZINC36833417 AKOS034259246 Z3812236321100111-58-7	-10.584

Table S7. Compound activity predictions using the sequential Keras/TensorFlow model of NN. Histograms showing the performance of Keras/TensorFlow in the activity prediction of CC chemokine receptor ligands. Data for CCR2 was included in the main manuscript (see Fig. 5). Ligand activities (pChEMBL) were divided into ranges (x-axis). The fraction of the dataset that was assigned to each activity range (in %) is included in brackets. Predicted activity values fell into three categories: overpredicted, underpredicted, and predicted correctly.

<table>
<tr><th></th><th>with inactive compounds</th><th>without inactive compounds</th></tr>
<tr><td>CCR1</td><td>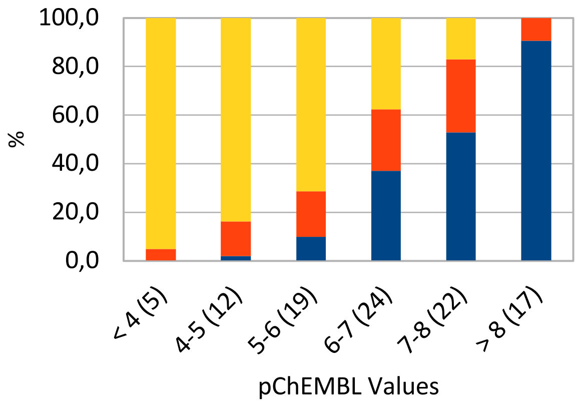
</td><td>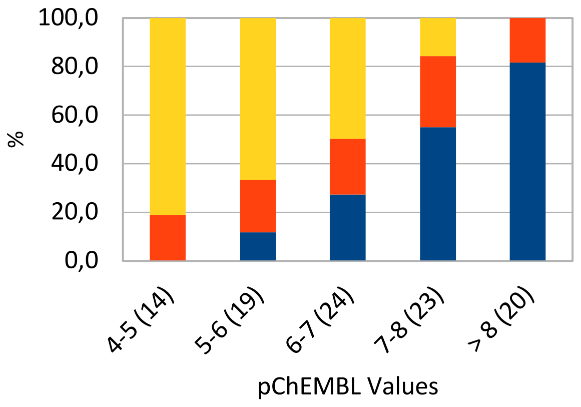
</td></tr>
<tr><td>CCR3</td><td>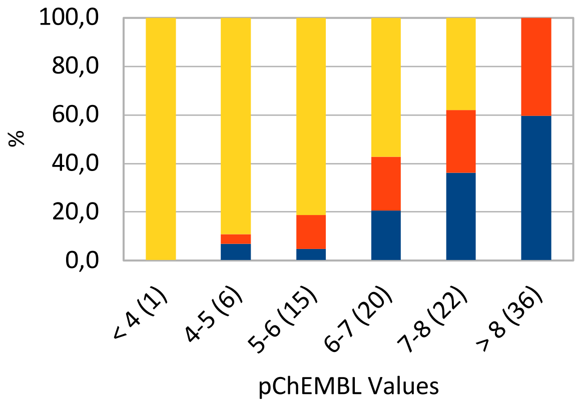
</td><td>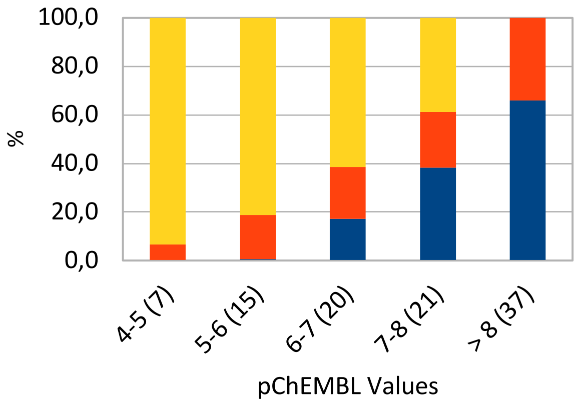
</td></tr>
<tr><td>CCR4</td><td>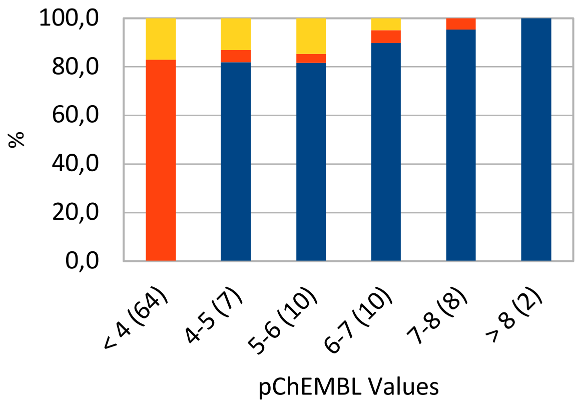
</td><td>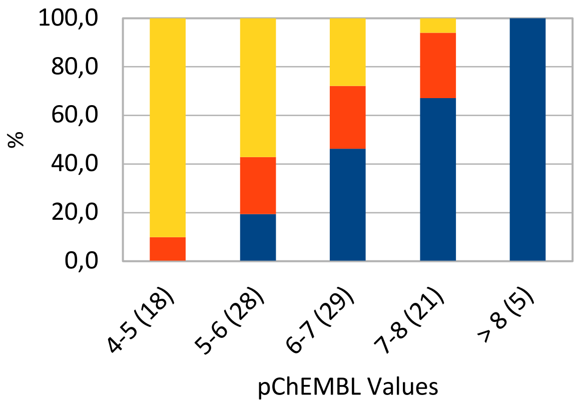
</td></tr>
</table>

<table>
<tr><td>CCR5</td><td>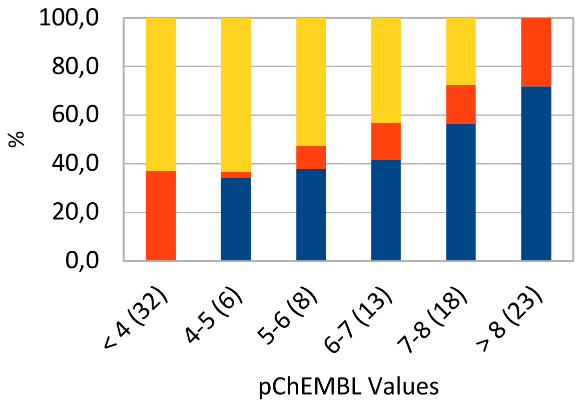
</td><td>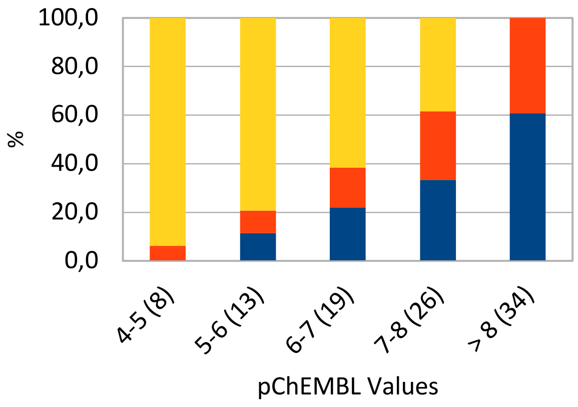
</td></tr>
<tr><td>CCR6</td><td>overpredicted
correctly predicted
underpredicted</td><td>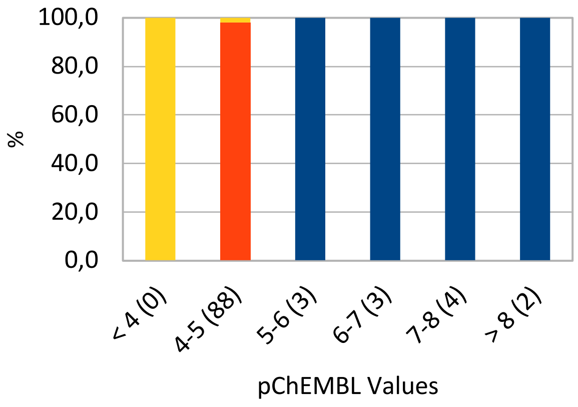
</td></tr>
</table>

Table S8. Compound activity predictions for CC chemokine receptors using LightGBM. Top – comparison of compound activities predicted by LightGBM compared to the know activity values (as defined by pChEMBL values), with (left) and without (right) inactive compounds included in the datasets. A perfect correlation line is included for comparison (red line). Data for CCR2 was included in the main manuscript (see Fig. 6).

<table>
<tr><th></th><th>with inactive compounds</th><th>without inactive compounds</th></tr>
<tr><td>CCR1</td><td>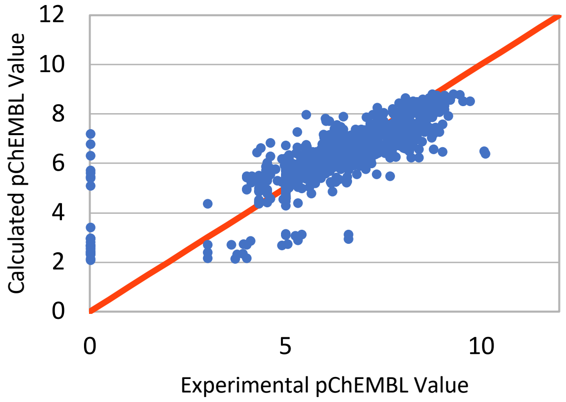
</td><td>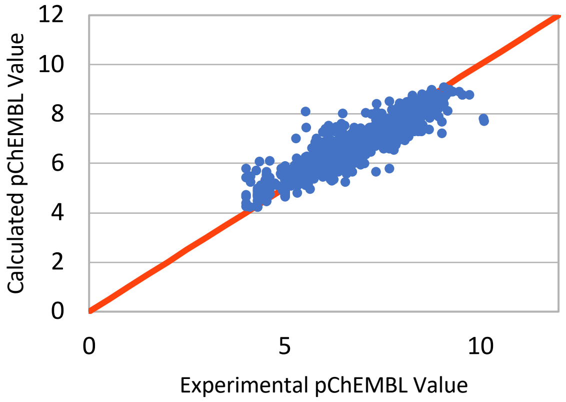
</td></tr>
<tr><td>CCR3</td><td>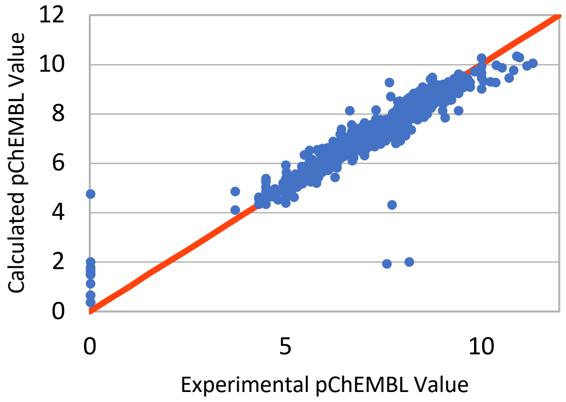
</td><td>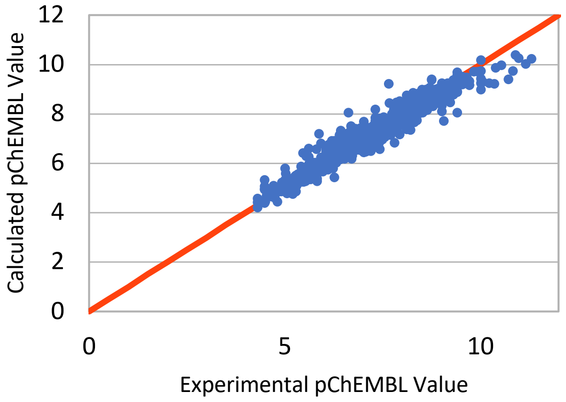
</td></tr>
<tr><td>CCR4</td><td>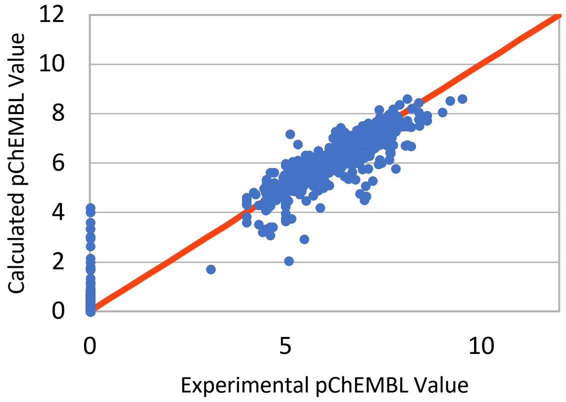
</td><td>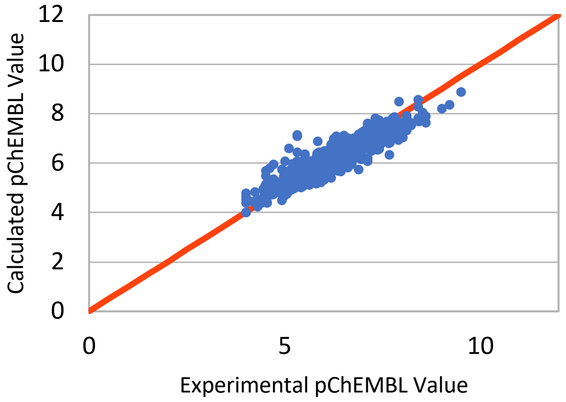
</td></tr>
</table>

<table>
<tr><td>CCR5</td><td>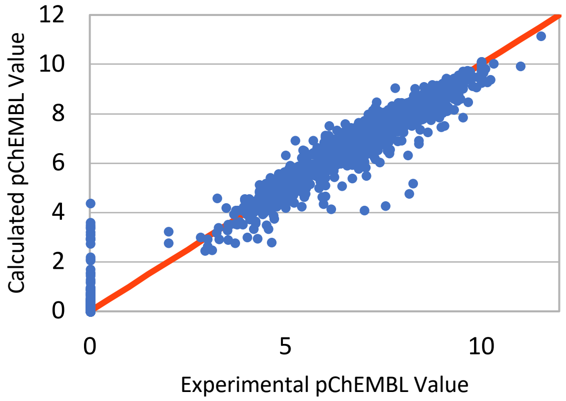
</td><td>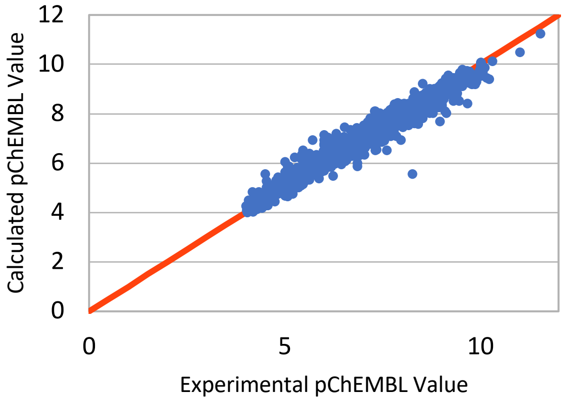
</td></tr>
<tr><td>CCR6</td><td>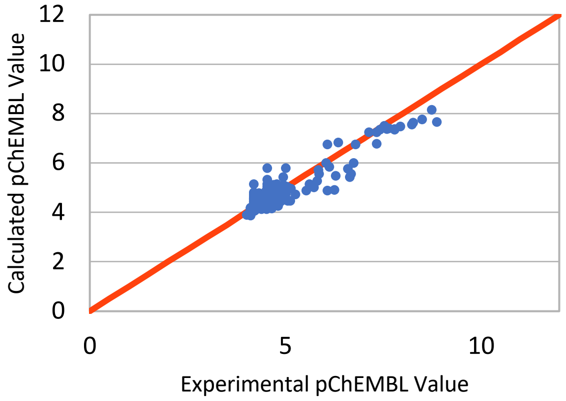
</td><td>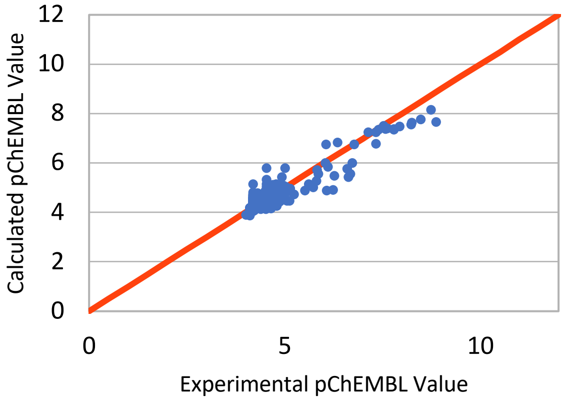
</td></tr>
</table>

Table S9. Compound activity predictions using LightGBM. Histograms showing the performance of LightGBM in activity prediction. Ligand activities (pChEMBL) were divided into ranges (x-axis). The fraction of the dataset that was assigned to each activity range (in %) is included in brackets. Predicted activity values fell into three categories: overpredicted, underpredicted, and predicted correctly. Data for CCR2 was included in the main manuscript (see Fig. 5).

	with inactive compounds	without inactive compounds
CCR1	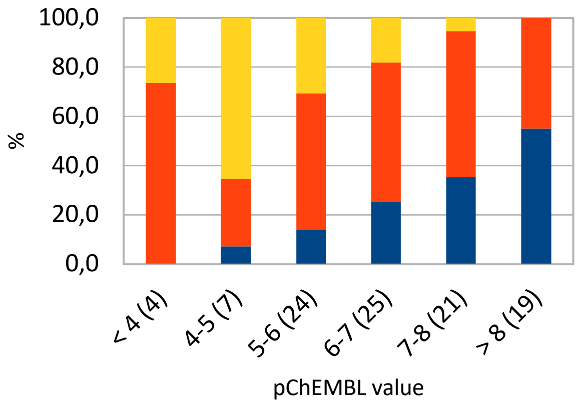	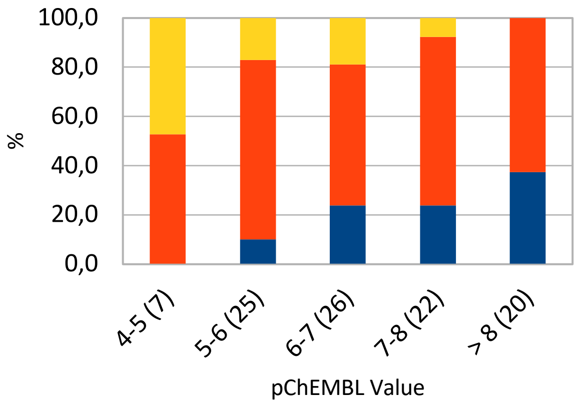
CCR3	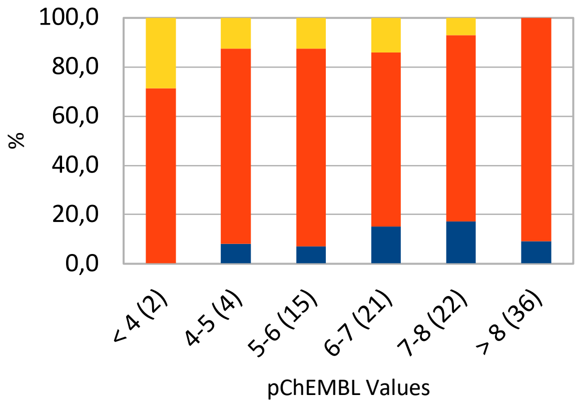	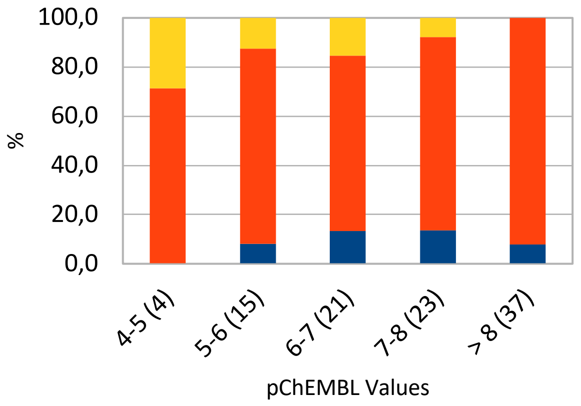
CCR4	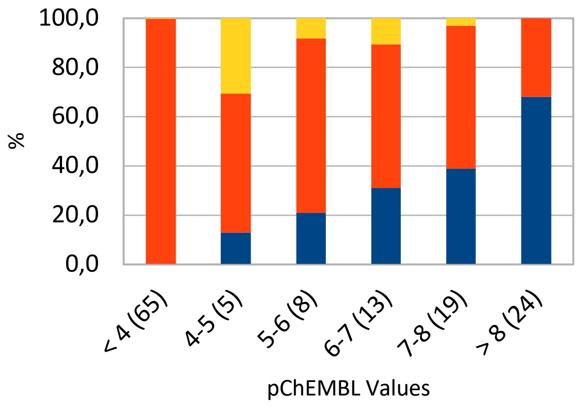	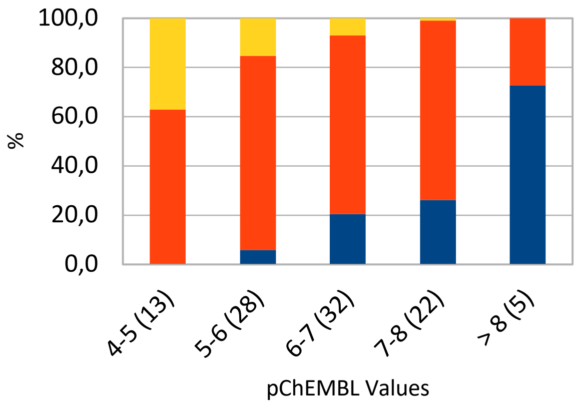

CCR5	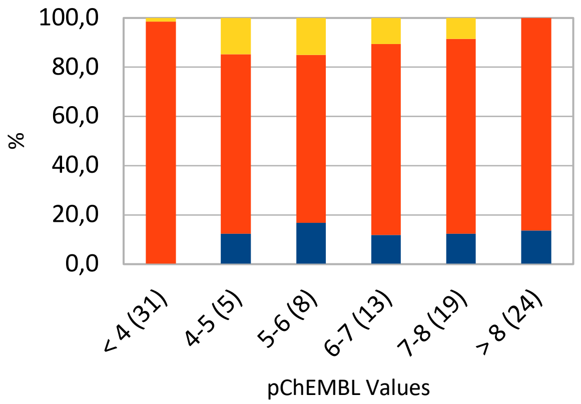100,0<br>80,0<br>60,0<br>40,0<br>20,0<br>0,0<br>%<br>< 4 (31)<br>4-5 (5)<br>5-6 (8)<br>6-7 (13)<br>7-8 (19)<br>> 8 (24)<br>pChEMBL Values	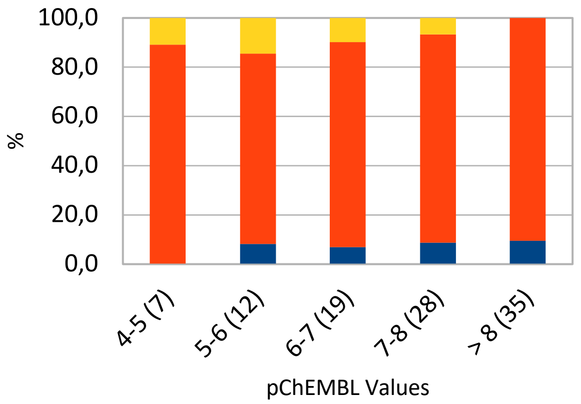100,0<br>80,0<br>60,0<br>40,0<br>20,0<br>0,0<br>%<br>4-5 (7)<br>5-6 (12)<br>6-7 (19)<br>7-8 (28)<br>> 8 (35)<br>pChEMBL Values
CCR6	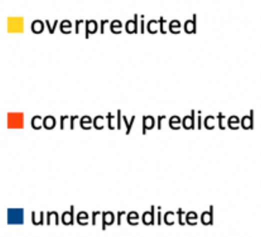overpredicted<br>correctly predicted<br>underpredicted	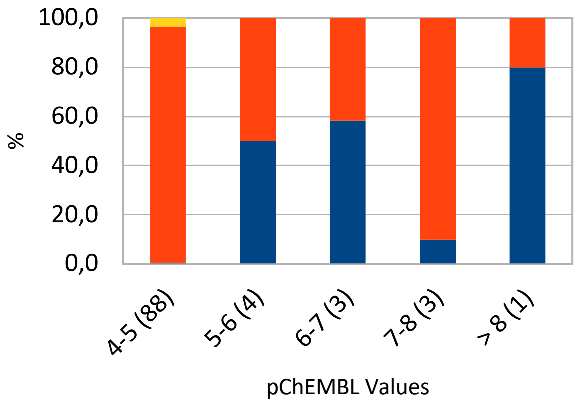100,0<br>80,0<br>60,0<br>40,0<br>20,0<br>0,0<br>%<br>4-5 (88)<br>5-6 (4)<br>6-7 (3)<br>7-8 (3)<br>> 8 (1)<br>pChEMBL Values

Table S10. CCR2 actives – Enamine compounds selected by SBVS assisted by LightGBM.

Rank	Cluster ID	SMILES	2D structure and its interactions	Enamine identifier	XP GScore
1	11	c1cccc(c12)[nH]c(n2)[C@@H](C)NC(=O)C[C@H](S3)C(=O)Nc(c34)cc(C(F)(F)F)cc4	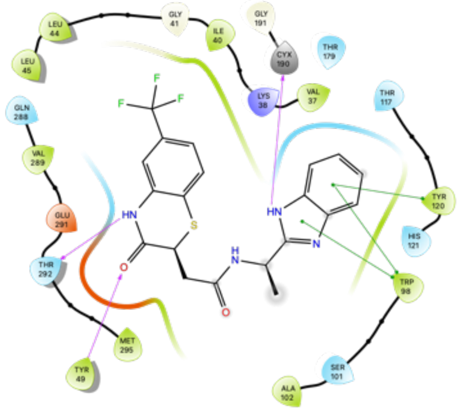	Z199951150*	-9.933
2	19	CO[C@@](C)(C(=O)[O-])C(=O)N[C@H]1CCN(C1)c2ncc(C(F)(F)F)cc2Cl	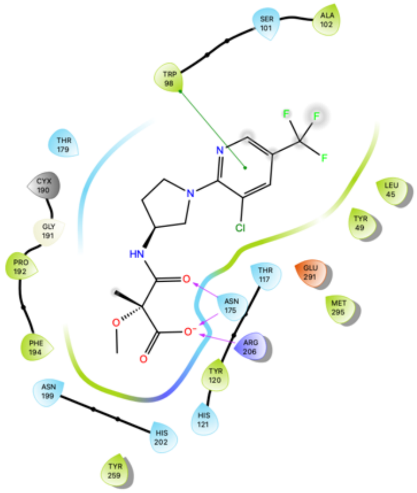	Z2607653088	-9.273
3	4	Oc1ccc(cc1)N2CCN(CC2)C(=O)Nc3ccc(C(F)(F)F)cc3	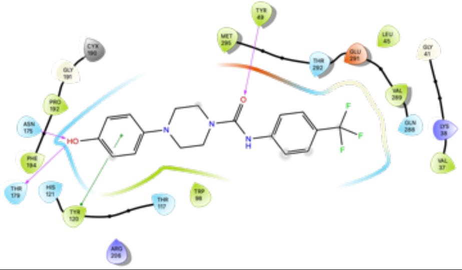	Z409952080 **	-8.541
4	18	CCNC(=O)N1CC[C@@H](C1)NC(=O)Cc2csc(n2)-c3ccc(C(F)(F)F)cc3	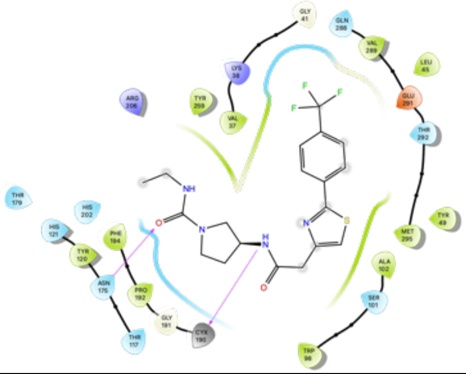	Z1420823261 ***	-8.371

5	11	c1cccc(c12)c(c[nH]2)C[C@H](C([O-])=O)NS(=O)(=O)c3ccc(Cl)c(c3)C(F)(F)F	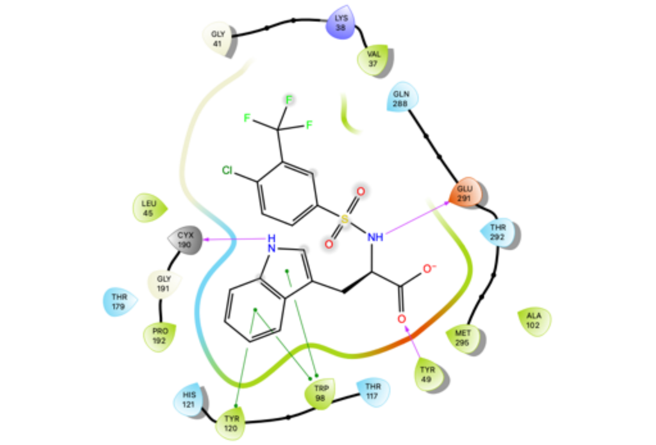	Z45637008	-8.346
6	11	c1cccc(c12)cc(c(=O)[nH]2)C[NH2+]C[C@@H](CC3)Cn(c34)cc(n4)C(F)(F)F	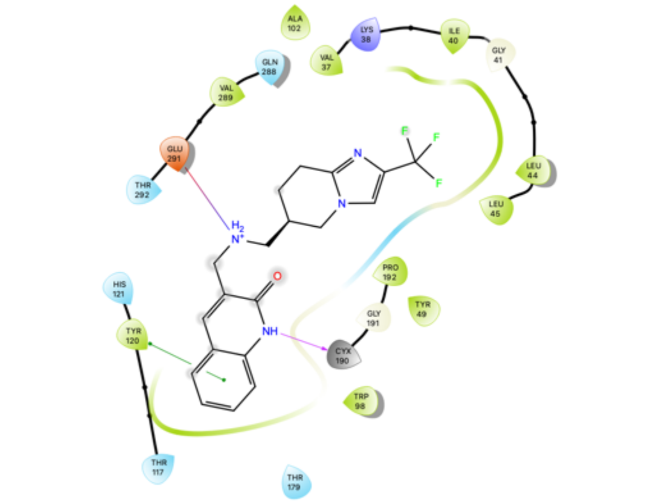	Z1757927604	-8.249

*Synonyms: N-[(1R)-1-(1H-benzimidazol-2-yl)ethyl]-2-[(2S)-3-oxo-6-(trifluoromethyl)-4H-1,4-benzothiazin-2-yl]acetamide; ZINC89904482

**Synonyms: SCHEMBL20291070; ZINC40097903; AKOS034291473; Z409952080; 4-(4-hydroxyphenyl)-N-[4-(trifluoromethyl)phenyl]piperazine-1-carboxamide

***Synonyms: ZINC84791026; (3S)-N-ethyl-3-[[2-[2-[4-(trifluoromethyl)phenyl]-1,3-thiazol-4-yl]acetyl]amino]pyrrolidine-1-carboxamide

Table S11. CCR2 actives – Enamine compounds selected by SBVS assisted by NN.

Rank	Cluster ID	SMILES	2D structure and its interactions	Enamine identifier	XP GScore
1	9	NC(=O)c1ccc(N2CCCC2)c(c1)NC(=O)CC[C@H](C3)C(=O)Nc(c34)cccc4	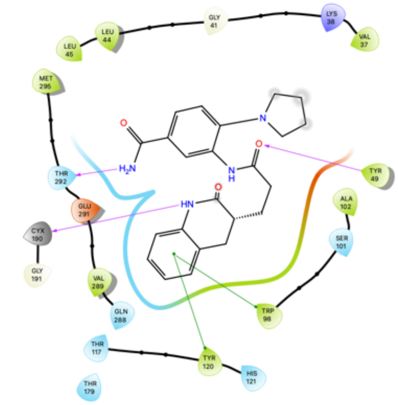	Z238859824*	-9.070
2	9	N1C(=O)CCc(c12)cc(c(F)c2)C(=O)NCC(=O)Nc3cccnc3	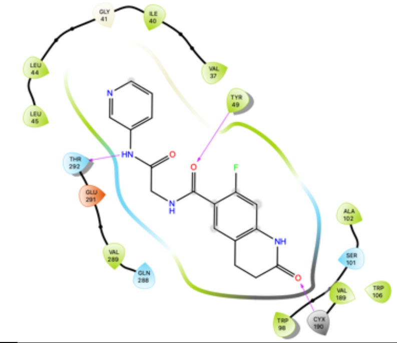	Z1447562795**	-8.582
3	9	C1COCCC1[C@@H](C)C(=O)Nc2ccc(OCC(=O)[O-])cc2C	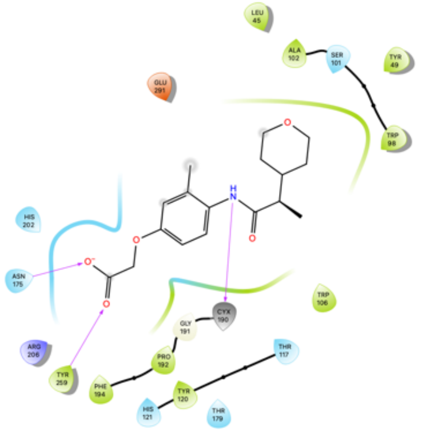	Z1991687578	-8.247
4	19	CCn(c(=O)[nH]1)c(=O)c(c12)ccc(c2)C(=O)N3CCO[C@@H](C3)C(=O)[O-]	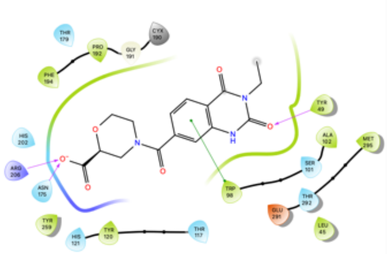	Z1445271329	-8.135

*Synonyms: ZINC23671255; 3-[3-[(3R)-2-oxo-3,4-dihydro-1H-quinolin-3-yl]propanoylamino]-4-pyrrolidin-1-ylbenzamide

**Synonyms: AKOS033352368; ZINC137145800; Z1447562795; 7-fluoro-2-oxo-N-[2-oxo-2-(pyridin-3-ylamino)ethyl]-3,4-dihydro-1H-quinoline-6-carboxamide

Table S12. CCR3 actives – Enamine compounds selected by SBVS assisted by LightGBM. Compounds in common with the NN-predicted subset of CCR3 actives were bolded.

Rank	Cluster ID	SMILES	2D structure and its interactions	Enamine identifier	XP GScore
1	9	Fc1cccc(c1)Cn2cc(ccc2=O)NC(=O)NC[C@@H]3CC[NH2+]C3	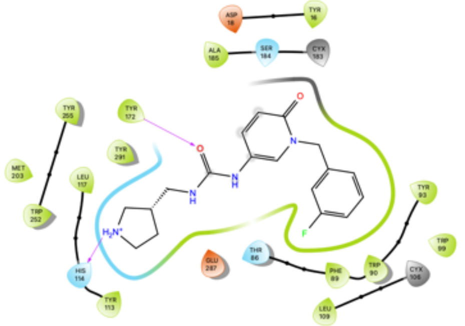	Z2997931543	-11.270
**2**	**10**	**Fc1cccc(F)c1C[N@@H+]2CCC[C@@H](C2)NC(=O)NCc(cc3)cc(n34)[nH+]cc4**	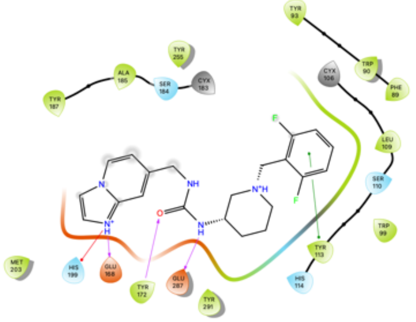	**Z1426245621**	**-10.942**
3	10	Fc1ccc(cc1)C[C@@H]2CCN(C2)C(=O)c3c[nH]c(n3)C4CC[NH2+]CC4	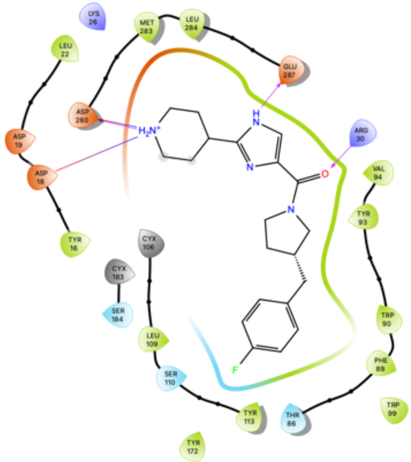	Z2764968046	-10.720
4	14	C[C@H](O)C[N@H+]1CCN(C[C@@H]1CC)C(=O)CCc(cc2)cc(c23)cccc3	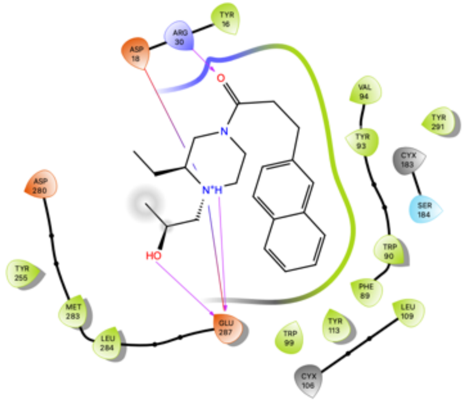	Z1274932994	-10.530

**5**	**14**	**C[C@H]1C[NH2+]CC[C@@H]1NC(=O)CCCc(cc2)cc(c23)c4c(C3)cccc4**	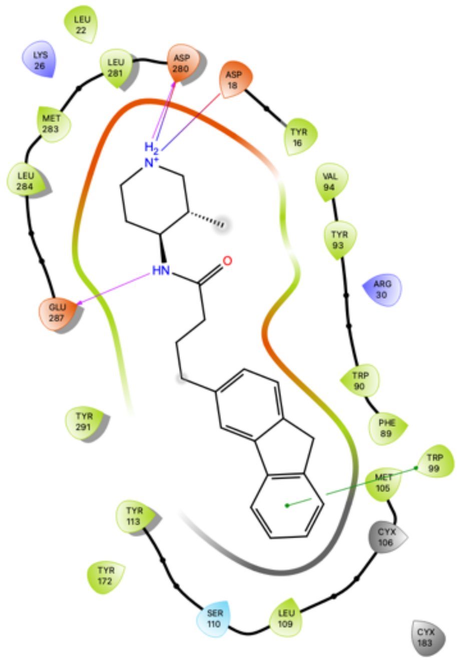	**Z2441027668**	**-10.515**
6	10	F[C@@H]1C[NH2+]CC[C@@H]1CC(=O)Nc2nc(C3CC3)c(s2)Cc4ccc(F)cc4	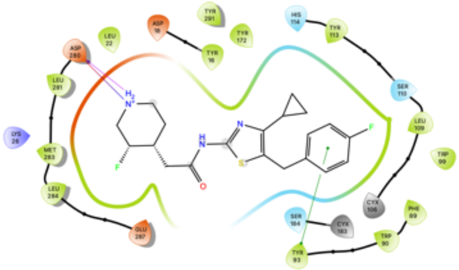	Z2606182917	-10.488
7	10	[NH3+]Cc1ccc(cc1)C(=O)N2CC[C@@H](C2)Cc3ccc(F)cc3	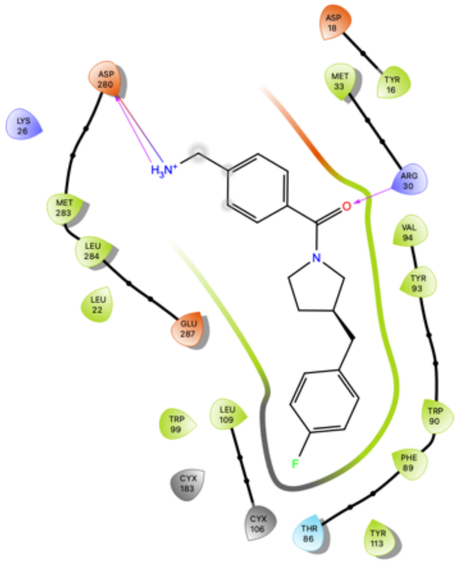	Z1450372344	-10.379

Table S13. CCR3 actives – Enamine compounds selected by SBVS assisted by NN.

Rank	Cluster ID	SMILES	2D structure and its interactions	Enamine identifier	XP GScore
1	14	c1cccc(c12)c(ccc2)NC(=O)[C@H](C)[N@@H+]3CC[C@H](CC3)Oc4cc[nH+]cc4C	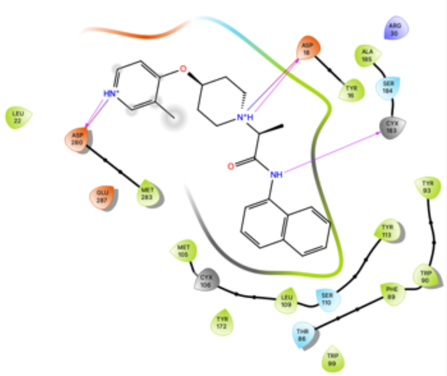	Z1912507172	-12.560
2	10	C[NH+](C)C[C@@H](O)CC(=O)N[C@H]1C[N@@H+](Cc2ccccc2)C[C@@H]1c3ccc(F)cc3	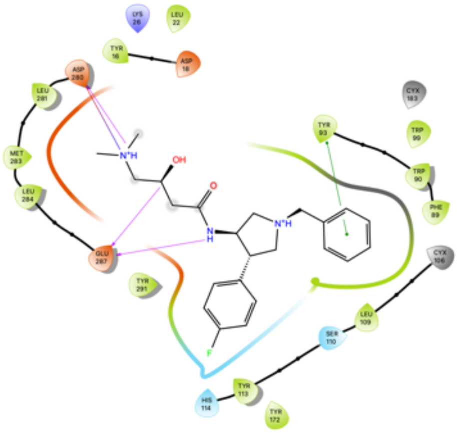	Z3584348903	-12.350
3	10	Fc1cccc(c1)NC(=O)CC[N@@H+]2CCCN(CC2)c3ccc(cn3)C(F)(F)F	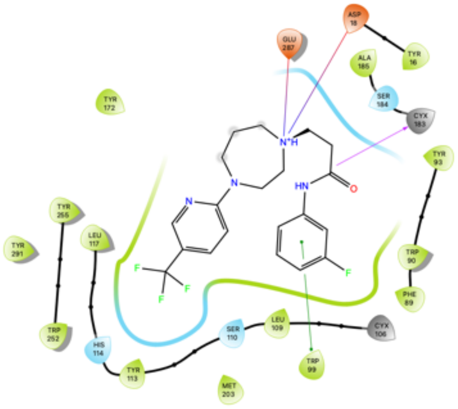	Z220095976	-12.271

4	10	Fc1cccc(F)c1C[N@@H+]2CCC[C@@H](C2)NC(=O)[C@H](CC3)Cc(n34)[nH+]cc4	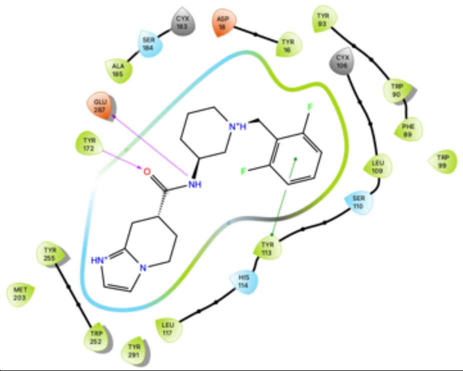	Z1489764574	-12.110
5	10	C[NH+](C)Cc1ccc(cc1C)NC(=O)CC[N@@H+](CC2)Cc(c23)cccc3	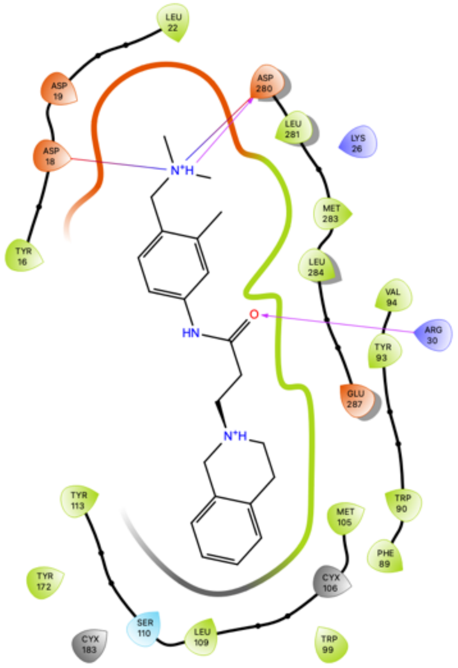	Z2358091793	-12.079
